# Spontaneous Formation of Solid Shell Polymeric Multicompartments at All‐Aqueous Interfaces

**DOI:** 10.1002/advs.202402592

**Published:** 2024-10-04

**Authors:** Francisca G. Perfeito, Sara Vilabril, Andreia Cerqueira, Mariana B. Oliveira, João F. Mano

**Affiliations:** ^1^ Department of Chemistry CICECO – Aveiro Institute of Materials University of Aveiro Aveiro 3810‐193 Portugal

**Keywords:** capsules, interfacial complexation, multicompartments, polyelectrolytes, soft materials

## Abstract

Multicompartmental capsules have demonstrated value in fields ranging from drug release, mimetics of artificial cells, to energy conversion and storage. However, the fabrication of devices with different compartments usually requires the use of toxic solvents, and/or the adaptation of technically demanding methods, including precision microfluidics and multistep processes. The spontaneous formation of multi‐core capsules resulting from polyelectrolyte complexation at the interface of a prototypic all‐aqueous two‐phase system is described here. The variation of polyelectrolyte concentration and complexation time are described as simple working parameters capable of driving the formation of compartments at different yields, as well as tailoring their morphology. The mild processing technology enables the encapsulation of animal cells, which are capable of invading capsule walls for specific processing conditions.

## Graphical abstract

The batch production of multi‐core polymeric capsules with unconventional architectures is reported. Separation phenomena happening at the polyelectrolyte complexation interface are hypothesized to drive the formation of the capsules. These materials are prepared in mild conditions compatible with cell encapsulation.

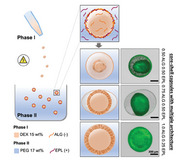

## 1 Introduction

Compartmentalization is a prevalent phenomenon observed in nature, normally related with the protection of different cargos (e.g., the embryo protection in amniotic sacs and eggs)^[^ ^1^ ^]^ and the enablement of function (e.g., a living cell holds multiple distinct compartments that carry out different biological tasks).^[^ ^2^, ^3^, ^4^ ^]^ While single compartment capsules have shown wide utility in bioengineering, biotechnology, and healthcare, with mRNA encapsulation in lipidic nanoparticles as one of the most recent examples with widespread application for vaccination,^[^ ^5^ ^]^ naturally occurring compartments usually organized in multiple core‐shell structures, often with non‐uniform architectonic and chemical features, such as in the example of living cells. For cell organelles, the different properties of protective compartments also enable a variety of functions through the maintenance of a variety of shapes, diffusion properties, as well as the content of encapsulated cargo.

Lab‐synthetized multicompartment objects most commonly comprise concentric multi‐shell^[^ ^6^, ^7^ ^]^ or uniformly divided spherical objects.^[^ ^8^ ^]^ Spatial versatility and monodispersion are usually obtained resorting to specially designed microfluidics setups^[^ ^9^, ^10^ ^]^ and, less often, to double emulsion batch strategies.^[^ ^11^, ^12^, ^13^ ^]^ Strategies explored so far make use of either polymer crosslinking strategies to produce spatially organized bulk hydrogel compartments (most often resorting to calcium‐alginate interactions),^[^ ^14^, ^15^ ^]^ or ensure the separation between an aqueous phase that generates the biomaterial shell compartment, followed by sacrificed oil or organic solvent phase.^[^ ^16^, ^17^, ^18^, ^19^ ^]^

The all‐aqueous processing of interfacially assembled membrane‐based materials has mainly addressed the fabrication of monocompartmental structures comprising core (liquid)‐shell (solid) spherical capsules and fibers/tubes^[^ ^20^, ^21^, ^22^, ^23^ ^]^ or even complex continuous shapes.^[^ ^24^ ^]^ Such materials are usually assembled at the stable interface of aqueous two‐phase systems (ATPS) or prototypical analogs, most commonly dextran/poly(ethylene glycol) solutions^[^ ^25^ ^]^ at certain concentrations.^[^ ^21^, ^22^ ^]^ Pickering emulsions formed by the same polymer pair have also been used as templates to prepare core‐shell capsules.^[^ ^26^, ^27^ ^]^ Applications of these structures have mostly comprised the encapsulation of cells for organoid and stem cells culture, as well as loading and release of macromolecules.^[^ ^4^, ^28^, ^29^, ^30^ ^]^ The spontaneous formation of separate compartments during the interfacial complexation of oppositely charged polyelectrolytes and polyelectrolyte‐nanoparticle systems in all‐aqueous setups was reported by Hann et al.^[^ ^31^ ^]^ in a phenomenon with analogy to the formation of double emulsions. The formed inner compartments, however, were completely liquid and did not enable the formation of structures with solid membrane multi‐compartments. Therefore, the formed inner compartment would be expected to be disrupted and washed off upon disturbing the equilibrated state of the formed structures with, for example, the addition of aqueous solvents. The latter are usually added to the formed capsules to remove unreacted reagents, or as a way to enable cell culture. The spontaneous formation of multi‐compartment structures with multiple solid components has not yet been reported.

Here, we report the spontaneous formation of multicompartment solid membrane core‐shell structures, with distinct architectures, using an all‐aqueous process. By keeping a close‐to‐immiscible all‐aqueous interface, and varying the concentration of two polyelectrolytes (alginate, ALG and ε‐poly‐L‐lysine, EPL) and time of interfacial complexation, multicompartmentalized close‐to‐spheric objects could be obtained in a simple and rapid procedure. The explored mild temperature, pH, and applied voltage enabled encapsulating viable animal cells in the generated materials. Depending on processing parameters, the preferential location of cells in the objects could be controlled. Specific work conditions led to complex shell morphologies that enabled cell migration, unattainable in single‐core capsules. The reported phenomena may find application in several fields of biotechnology and bioengineering targeting widespread applications including cell‐based applications, development of unconventional drug retention and delivery systems, or the establishment of miniaturized separation systems.

## 2 Results and Discussion

**Figure 1:**
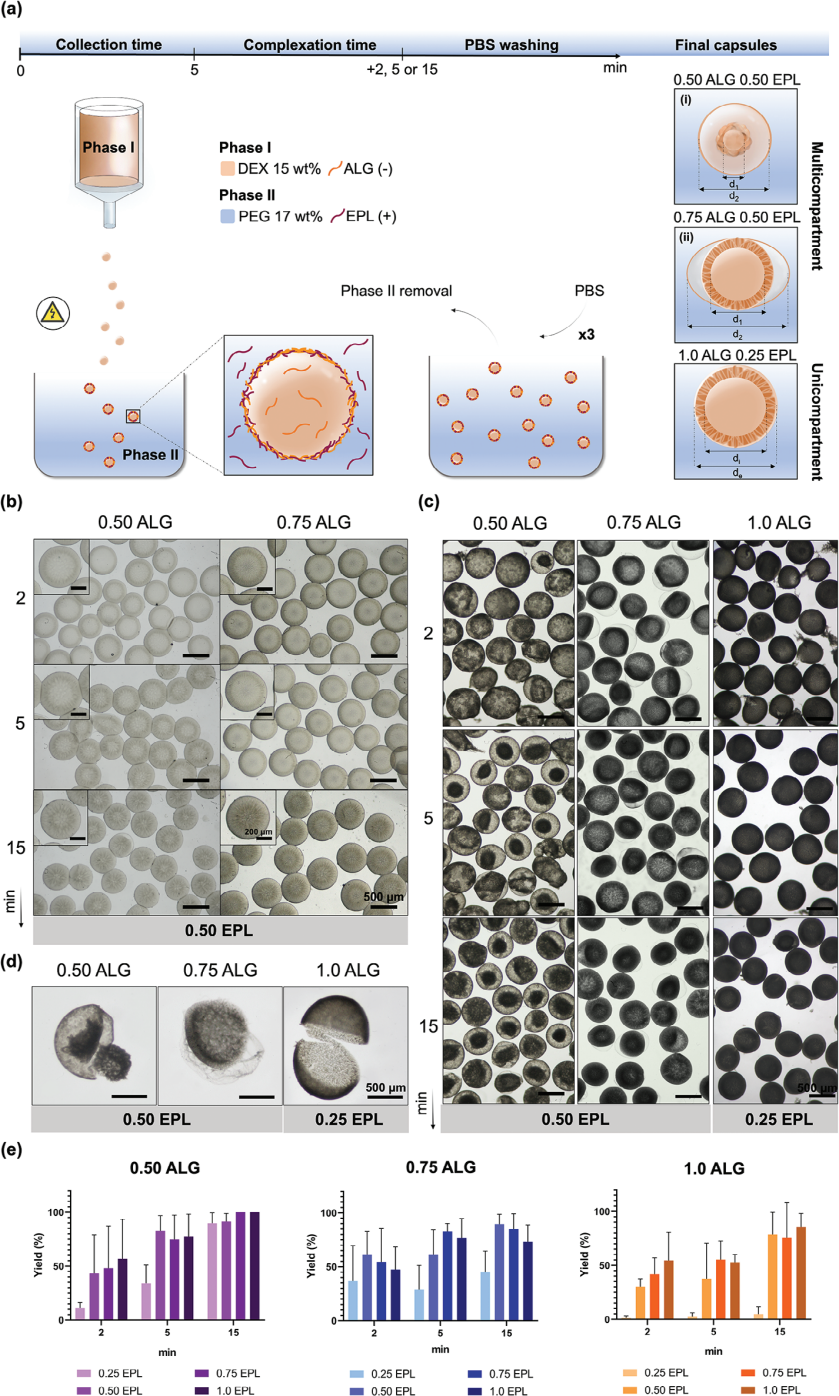
Schematic representation of complexation mechanism for the formation of uni‐and multicompartment capsules. a) Stable capsules by interfacial complexation between negatively charged alginate (ALG) and positively charged ε‐poly‐L‐lysine (EPL) at the precursor ATPS interface composed of poly(ethylene glycol) (PEG) and dextran (DEX). b) Optical micrographs of uni‐ and multicomparment capsules before PBS washes. c) Optical micrographs of the capsules upon PBS washing. d) Microscopy images of uni‐ and multicomparment capsules’ cross sections obtained for 5 min of collection and further 5 min of complexation for 0.50 wt.% ALG + 0.50 wt.% EPL, 0.75 wt.% ALG + 0.50 wt.% EPL and 1.0 wt.% ALG + 0.25 wt.% EPL. e) Yield analysis in percentage for all the conditions tested for three independent experiments (*n* > 40 capsules analyzed per assay). Data is shown as mean ± standard deviation (*n* = 3 independent experiments).

The formation of liquid‐core capsules based on the interfacial complexation of two oppositely charged polyelectrolytes – EPL and ALG – was achieved using an electrohydrodynamic spraying apparatus and spraying conditions, analogously to previous reported by Vilabril et al.,^[^ ^22^ ^]^ and as schematically represented in **Figure** **1**a. In this system, when the dispersive phase – Phase I – (alginate + dextran, ALG + DEX) is immersed into a volume of the continuous phase – Phase II – (ε‐poly‐L‐lysine + poly(ethylene glycol), EPL + PEG), a spontaneous solid membrane forms at the liquid‐liquid interface. Upon contact, ALG + DEX solution is entrapped inside of a forming membrane, which is expected to allow the rapid diffusion of the small EPL molecules into the ALG + DEX solution; this molecular dynamic was corroborated by membrane growth studies and calculation of partition coefficients at the interface of tubes made using a similar system as the ones explored here.^[^ ^21^ ^]^ In partition studies, the PEG/Dextran system was mixed in the presence of EPL or alginate; while alginate tended to completely partition to the dextran phase, indicating a poor affinity for the PEG phase, EPL tended to partition toward both phases, with predominance to the dextran phase. Here, the use of varying concentrations of polyelectrolytes enabled the spontaneous formation of capsules with different compartments and morphologies. All capsules kept a continuous spherical non‐ruptured membrane that remained in contact with the outer medium (Figure 1b), even after washing with phosphate buffered salide (PBS) (Figure 1c). By mixing varying concentrations of ALG – 0.50 to 1.0 wt.% – in a 15 wt.% DEX solution – with a 0.25 to 1.0 wt.% EPL in a 17 wt.% PEG solution and exploring the polyelectrolytes complexation for 2, 5, and 15 min, capsules with distinct morphologies and compartments were spontaneously obtained after washing (Figure 1c). Overall, two major types of capsule morphologies were obtained by varying the concentrations of polyelectrolytes, as well as reaction time for interfacial complexation: (i) capsules with a compact inner compartment with a darker and shriveled appearance (e.g., 0.50 wt.% ALG + 0.50 wt.% EPL) and (ii) capsules with an outer transparent membrane that partially surrounds an inner capsule with apparent higher compactness were formed (e.g., 0.75 wt.% ALG + 0.50 wt.% EPL). The analysis of cross‐sections of capsules, as shown in Figure 1d, processed with 0.50 wt.% EPL and increasing concentrations of ALG corroborated that unicompartment capsules were formed by a single solid membrane, while different types of multicompartments could be obtained for decreasing concentrations of ALG. Among all studied conditions, most conditions processed from 0.25 wt.% EPL and 1.0 wt.% ALG showed very low yields of formation of multicompartments (Figure 1e). Overall, increasing times of complexation also correlated with increasing yields of formation of multicompartmental capsules, with highest efficiencies observed for the highest EPL concentration (Figure 1e).

**Figure 2:**
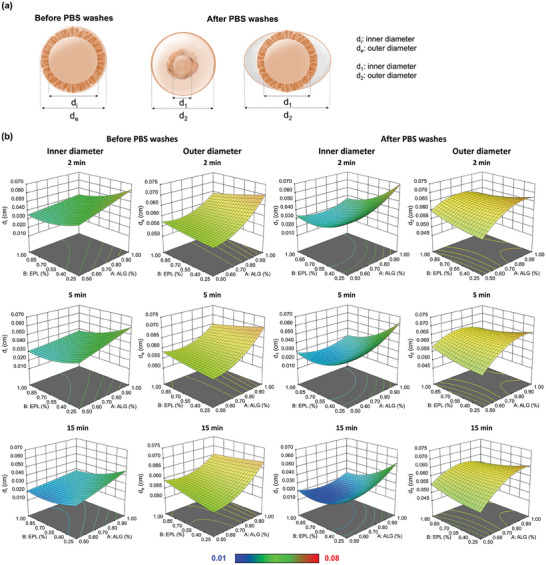
3D surface response maps. a) Scheme of the different capsules, being d_e_ and d_2_ the outer membrane diameter in uni‐ and multicompartment capsules, respectively; and d_i_ and d_1_ corresponding to the inner membrane diameter for the uni‐ and multicompartment capsules, respectively, both before and after PBS washes. b) plotting of 3D surface response maps for all the conditions tested before and after PBS washes. For conditions with yields of multicompartment formation after washing below or ≈10%, we considered d_1_ = d_2_.

Washing the complexed capsules with PBS is expected to disrupt the quasi‐equilibrated state of the prototypical ATPS. Although the formation of multiple compartment could only be observed after washing steps, we assessed whether a tendency for different membrane organization could be detected for different conditions before washing (Figure 1b; Figures S1–S3, Supporting Information). Overall, the outer diameter of pre‐washing capsules obtained for all conditions was similar, varying from average values of ca. 560 to 690 mm, with a slight increase for higher alginate concentrations (**Figure** **2**). The high viscosity of the alginate solutions is known to create adhesion forces between the extruded polymer and the apparatus needle, which is here hypothesized to have led to slower dripping rates and therefore larger microcapsules, as previously reported.^[^ ^32^ ^]^ Higher times of complexation tendentially led to the formation of thicker precursory membranes (Figures S4–S6, Supporting Information). This corroborates previous studies in fibers that showed a time‐dependent diffusion and concomitant complexation of EPL through the alginate‐containing core of the droplets.^[^ ^21^ ^]^ Two different nomenclatures where attributed to the membranes before and after washing: d_e_ and d_i_ refer to the inner and outer diameter of unicompartmental capsules; d_2_ and d_1_ correspond to the outer diameters of the distinct compartments in multicompartmental capsules. A faster growth of d_e_/d_i_ ratio was observed in droplets with lower alginate concentration. This suggests that EPL can diffuse more easily in matrices with lower density (i.e., lower alginate concentration). Multicompartment capsules were also obtained by airspray equipment (Figure S7, Supporting Information), proving the versatility of the phenomenon.

The disruption of the quasi‐equilibrated DEX/PEG interface through washing with PBS led to morphological changes in all capsules (Figure 1c; Figure S1–S6, Supporting Information), with only a restricted number of conditions remaining unicompartmental. In general, the maintenance of the outer diameter was observed for all processing conditions after washing and equilibration in PBS (Figure 2). When increasing the complexation time, the capsule's diameter tends to increase relatively to the most inner compartment diameter as proven by the d_2_/d_1_ ratio values (Figure S8, Supporting Information). An increase in ALG concentration from 0.50 to 1.0 wt.% led to an increase in the average capsule's external diameter from ca. 545 to 640 mm (Figure 2). Interestingly, the external capsule's diameter tends to be maintained or go through a slight increase when compared to pre‐washed structures (Figure 2).

**Figure 3:**
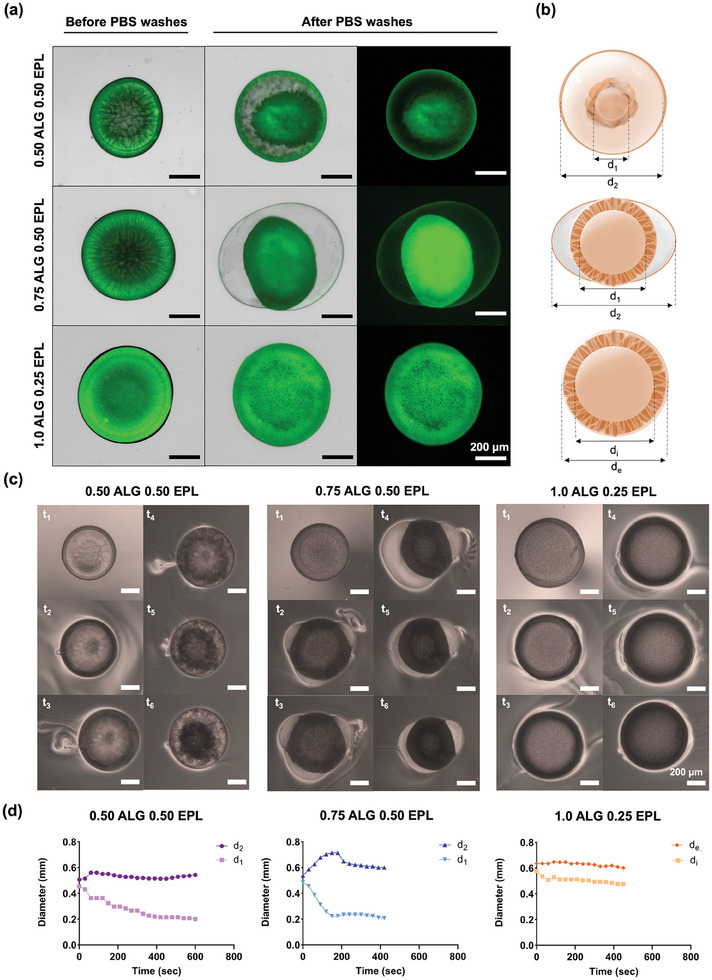
Characterization of uni‐ and multicompartment capsules produced in all‐aqueous environments. a) Optical and fluorescence micrographs of uni‐ and multicompartment capsules with FITC labeled EPL obtained for 5 min of collection and a further 5 min of complexation, before and after PBS washes. b) Scheme of the hypothesized mechanism of the formation of uni‐ and multicompartment capsules. c) Optical micrographs taken during the PBS washes to obtain the final uni‐ and multicompartment structures. d) Diameter variation with time through the PBS washes of both outer membrane (d_e_;d_2_) and inner membrane (d_i_;d_1_) of the uni‐ and multicompartment capsules.

**Figure 4:**
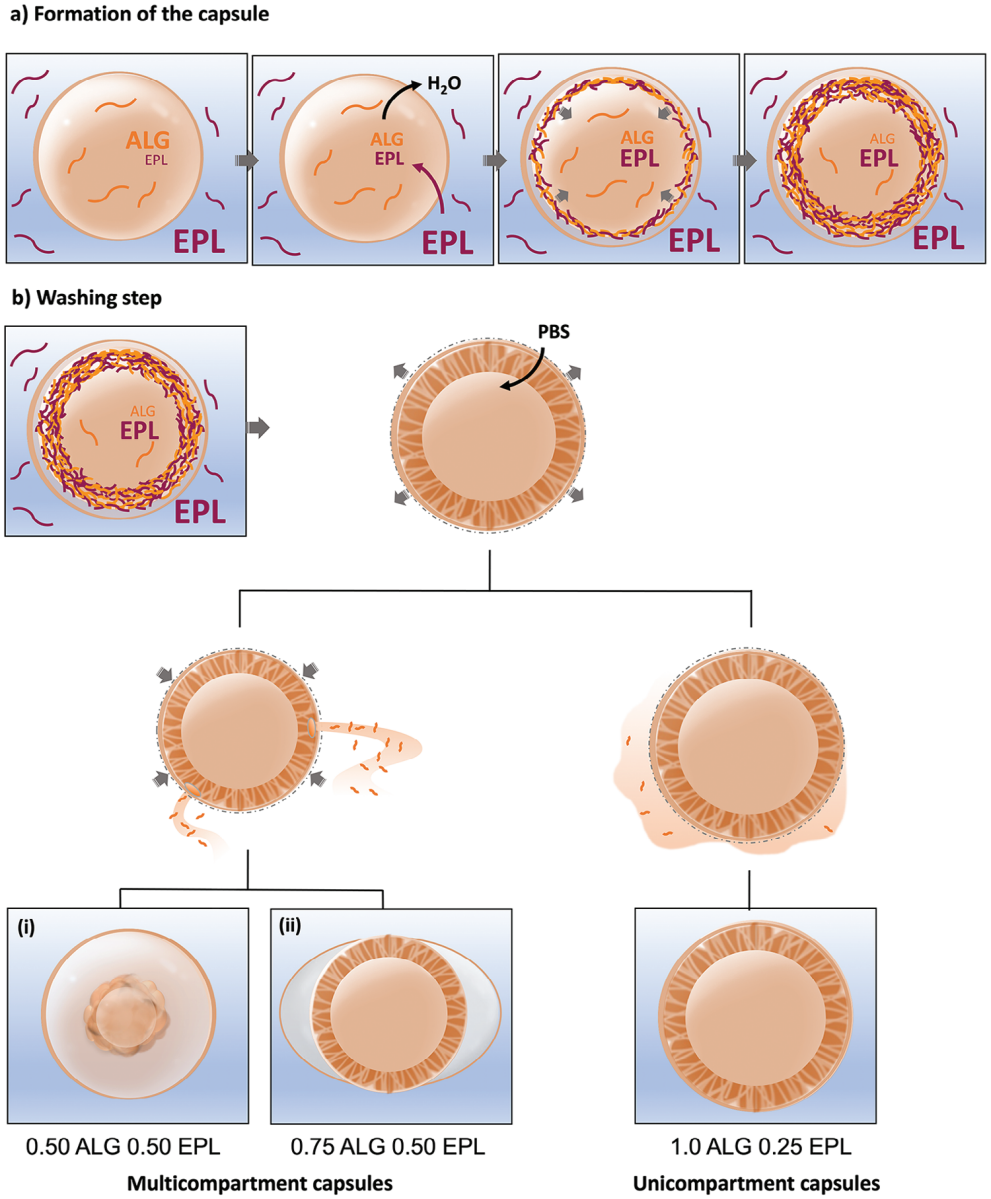
Schematic representation of the formation of the uni‐ and multicompartment capsules.

EPL was labeled with a fluorescent dye – fluorescein isothiocyanate (FITC) – to track its location during the formation of capsules, as well as its presence in the remaining structures after washing. Three formulations representative of distinct capsular morphologies were selected for analysis (**Figure** **3**a). As expected, unicompartment capsules (1.0 wt.% ALG) showed a dense EPL‐rich membrane, restricted to the boundary with the surrounding medium. In opposition, in the capsules with multiple compartments (0.50 and 0.75 wt.% ALG), two defined structures also containing EPL could be identified. The presence of EPL after washing suggests that both stable structures that compose multicompartment capsules are made of EPL complexed with ALG, analogously to the membrane of the unicompartmental capsule.^[^ ^22^ ^]^ To better understand the formation of different capsules until an equilibrated state after washing, the formation and washing process was observed using an optical microscope. Variations in the outer capsule membrane (for unicompartments), as well as for the outer and inner diameters (for multicompartments of types (i) and (ii)) showed that the outer compartments of unicompartment capsules and type (i) capsules remained stable in size after the washing step procedure. A time‐dependent shrinkage of the inner compartments was observed for multicompartment capsules of type (i) and (ii), with a faster tendency for stabilization, ≈150 s, for type (ii) capsules (Figure 3b,c). The latter probably correlates with the maintenance of a hollow semi‐inflated core, as observed in Figure 1d. Exceptionally, the external diameter of type (ii) capsules tends to swell right after addition of PBS. Unlike for unicompartment capsules, as observed in Figure 3a–c and Movie S3 (Supporting Information), for both types of multicompartment capsules the formation of breaches that enabled the draining of the liquid content of the capsules was observed after the addition of PBS (Figure 3b, Movies S1 and S3, Supporting Information). The swelling of the thin outer membrane of type (ii) capsules may be explained by the entrance of water to the inner core of the capsule, leading to the expansion of the thin – as suggested by the light shade in optical microscopy and low fluorescence detected for EPL ‐, yet expectedly flexible membrane. The diameter of the inner core of type (i) capsules tends to steeply decrease over time, when compared to type (ii) membranes. This may be related to the complete detachment of the inner compartment from the outer initially complexed membrane, which leads to its rupture and shrinkage, losing its hollow and sphere‐like nature. We hypothesize that the formation of multicompartment capsules after washing may be explained in several steps, as depicted in **Figure** **4**:

Step 1. Formation of a primary complexed membrane right after the contact of the droplet (ALG + DEX) and the external continuous bath (EPL + PEG).
Step 2. Rapid loss of water from the droplet due to the high EPL molar concentration present in the external continuous bath solution. This is expected to generate a volume loss in the droplet phase, rendering a secondary region of complexation, generating a secondary solid membrane.
Step 3. Growth of the secondary (inner) membrane due to the availability of alginate (from the inner core) and EPL, which is expected to enter through the thin permeable primary membrane, accordingly to partition coefficient studies performed in our previous study.^[^ ^21^ ^]^
Step 4. Maintenance of the first formed membrane, or its slower growth due to the low accessibility to alginate, which is hypothesized to stay restricted to the inner formed compartment (Figure 3a,b).
Step 5. The washing steps with PBS apparently lead to an initial entrance of water in the capsules, which is suggested by the increase in the overall external diameter capsules (i) and (ii). After some seconds (Movies S1 and S2, Supporting Information), perforations in the membrane are generated and lead to the expelling of the inner content of the capsules (probably ALG + DEX). The perforations are probably generated by localized mechanical stress caused by the stretching of more fragile regions of the complexed membranes, caused by the entrance of water in the capsule system. Its combined effect with the expelling of capsules’ inner content leads to the deflation of the inner compartments for both capsules (i) and (ii).

To better understand the dependency of membrane formation on an osmotic phenomenon, 0.25 m calcium chloride (NaCl) was added to PBS solutions with dissolved PEG and EPL (phase II) prior to atomization and complexation. Capsule formulations prepared with 0.75% ALG were studied with varying concentration of EPL. The increase in ionic concentration in the outer part of the membrane interface was expected to lead to a higher and possibly faster release of water for the dextran and alginate compartment. We hypothesized that this could lead to a more extensive shrinkage of the inner droplet, rendering a secondary interfacial complexation region that would be more distant from the primary membrane, when compared to conditions prepared with lower ion concentration in the bath phase. We observed that the addition of salts to the outer phase led to the formation of clearly visible separated thin outer membranes in the multicompartment capsules. Unlike for control conditions, in which PEG and EPL were dissolved only in PBS – and the clear visualization of the inner and outer membranes was only detectable after washing ‐, when additional 0.25 m NaCl was added, the separation between the inner and outer compartments was visible before the washing step (Figure S9, Supporting Information), indeed suggesting a more extensive separation of both interfaces right from the beginning of the formation of the capsules. The morphology of post‐washing capsules could not be analyzed because the outer membrane of capsules formed in the presence of additional 0.25 m NaCl tended to rupture after the washing step with PBS, suggesting that the addition of NaCl may have lowered the efficiency of polyelectrolyte complexation. In addition, the effect of possible pH variations in the phases was also studied. However, within pKa values of both polyelectrolytes that enabled the formation of stable capsules – which exclude the use of EPL at pH values as 9 ‐, significant morphological alterations could not be detected in the overall number of compartments and their spatial organization (Figure S10, Supporting Information).

The stability of the capsules was kept with unaltered morphology after 6 months of storage in PBS at room temperature (Figure S11, Supporting Information). When immersed in a PBS solution at pH 5, the integrity of the compartments was maintained, while at pH >9, multicompartment capsules swelled until becoming entirely invisible under optical microscopy after 47 min for 0.75 wt.% ALG + 0.50 wt.% EPL capsules, and after ≈20 min for the 0.50 wt.% ALG + 0.50 wt.% EPL capsules. These tendencies corroborated the observations reported by Gonçalves et al.^[^ ^21^ ^]^ for continuous fibers prepared using a similar system, which were hypothesized to be mainly mediated by protonation states of polyelectrolytes around their pKa values. We also experimentally determined the pKa values of the polyelectrolytes dissolved in PBS used to prepare multicompartiment capsules. The obtained ranges were 8.8‐9.7 and 4.3‐4.5 for EPL and alginate, respectively, which reinforces the role of polyelectrolyte protonation in the stability of the formed capsules. Here, the exposure of multicompartment capsules to solutions with varying pH values enabled the selective destruction of outer layers and the preservation of the inner cores of the capsules for times of exposure between 600 and 1000 s (Figure S12a,b, Supporting Information), opening the possibility for the achievement of complex stimuli‐responsive cargo release. Mechanical robustness was explored as a parameter to enable the selective rupture of the outer membranes. Uni‐ and multicompartment capsules were exposed to centrifugal forces ranging from 150 to 5525 *g* (Figure S12c, Supporting Information). While exposure to 150 *g* for 5 min led to rupture of the outer membrane, higher rotational forces as high as 5000 *g* were not sufficient to rupture of the inner compartment. The outer membranes of 0.50 wt.% ALG + 0.50 wt.% EPL and 0.75 wt.% ALG + 0.50 wt.% EPL capsules could also be disrupted by micropipetting – using a micropipette tip with 429 mm – while preserving the inner cores (Figure S12d, Supporting Information).

**Figure 5:**
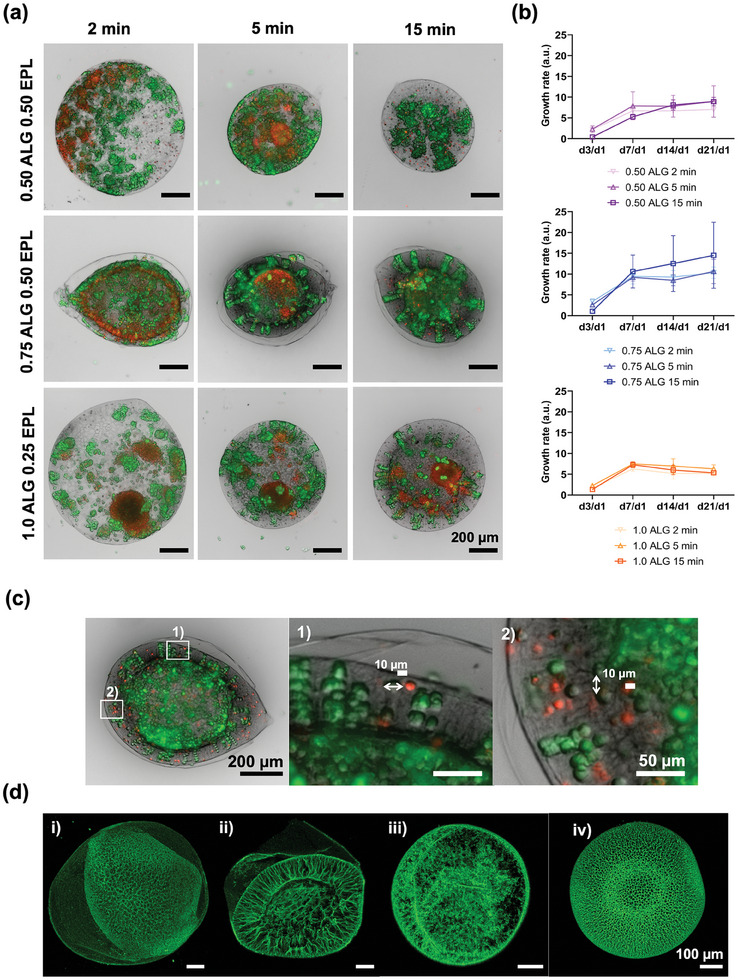
Encapsulation of cells. Uni‐ and multicompartment capsules are produced and washed with Dulbecco's phosphate buffered saline (DPBS) after 5 min of collection and further 2, 5, and 15 min of complexation, placed on well‐plates with appropriate cell culture medium. Cells start to adhere through the all‐aqueous capsules after several days of culture. a) Live/Dead micrographs of uni‐ and multicompartment capsules for 0.50, 0.75, and 1.0 wt.% ALG after 7 days of culture. Staining – green: calcein‐AM (live), red: propidium iodide (PI, dead). b) Growth rate of encapsulated cells. Average values, and error bars show standard deviation. c) Close‐up micrograph of the channels formed in the 0.75 wt.% ALG capsule's membrane after the DPBS washing step. (d) Confocal microscopy images of capsules. i) Top view of a capsule prepared with 0.75 wt.% ALG + 0.50 wt.% EPL, for 5 min complexation time, ii) cross‐section of a capsule prepared with 0.75 wt.% ALG + 0.50 wt.% EPL, for 5 min complexation time, iii) cross‐section of a capsule prepared with 0.50 wt.% ALG + 0.50 wt.% EPL, for 5 min complexation time, iv) top view of a capsule prepared with 1.0 wt.% ALG + 0.25 wt.% EPL, for 5 min complexation time.

Liquid‐core capsules formed at the interface of separating all‐aqueous systems have been previously suggested by us and others for the encapsulation of living cells.^[^ ^31^, ^33^ ^]^ In unicompartment capsules, different types of adherent cells – including adipose stromal cells and the MC3T3‐E1 cell line – tended to mostly remain in the center of the capsules. Over time, both under static or stirred culture, cells tended to form self‐organized aggregates. Although studies in tubes formed by similar processes indicate that a small fraction of cells is retained at the interface complexed membrane,^[^ ^21^ ^]^ no significant cell adhesion and cell spreading at the capsule wall was observed for structures with single compartments.^[^ ^21^, ^22^ ^]^ Here, cells were mixed in the droplet phase for the production of capsules in conditions that led to specific multicompartment morphologies in cell‐free processes, as shown in the aforementioned results (**Figure** **5**a,c). The effective entrapment of cells in the capsules was studied by co‐localizing recently washed capsules with cells pre‐labeled with a fluorescent lipophilic dye. Overall, all cell cargo remained inside the formed capsules, and no free cells could be found in the surrounding washing solution (Figure S13, Supporting Information). In general, all conditions led to similar cellular growth rates (Figure 5b), with apparent stagnation at day 7. Exceptionally, the multicompartment condition 0.75 wt.% ALG + 0.50 wt.% EPL with 5 min complexation enabled an increasing cell growth rate up to ay 21 of culture (Figure 5b; Figure S14a, Supporting Information). The area occupied by living and dead cells over time in different capsules was also similar patterns for all experimental conditions (Figure S14d, Supporting Information). Higher areas occupied by living cells were detected for day 21 for all conditions, as well as the presence of dead cells (≈50% in relative area), mostly related to the formation of necrotic cores (Figure S14d, Supporting Information). A comparison with standard encapsulation in calcium‐crosslinked alginate continuous matrices (Figure S15, Supporting Information) showcased the ability of different multicore architectures to enable peculiar patterns of cell organization, in capsules with size and shape stability over time. For example, on day 1 of cell culture 0.50 wt.% ALG + 0.50 wt.% EPL, 0.75 wt.% ALG + 0.50 wt.% EPL, and 1.0 wt.% ALG + 0.25 wt.% EPL capsules presented diameters of 791 µm ± 53.9 µm, 756 µm ± 70.3 µm, and 1095.7 µm ± 55.76 µm, respectively. As for day 21, these values varied to 813 µm ± 45.6 µm, 721 µm ± 44.9 µm, and 1058 µm ± 79.3 µm, respectively, showcasing the ability of these capsules to withstand significant cell growth without significant variations in architecture (Figure 5; Figure S14, Supporting Information). In conventional continuous alginate hydrogels, L929 cells were capable of proliferating (Figure S15, Supporting Information). Unlike for core‐shell capsules, the swelling of the structure was observed after one day of cell culture, with a high increase in diameter. Also, cells were randomly distributed in the matrix, without the ability to self‐assemble even for 14 days of culture (Figure S15, Supporting Information).

The first assessed aspect was related to the ability of the capsules to maintain their morphology upon the insertion of living cells in the droplet phase. As previously reported,^[^ ^22^ ^]^ unicompartment capsules complexed for short periods (2 and 5 min) kept their unicompartmental features, mostly leading to the time‐dependent formation of multiple cellular aggregates in the interior of the capsules (Figure S14c, Supporting Information). However, longer times of complexation – 15 min – led to the unexpected formation of an initially cell‐free membrane, which was invaded by cells after 7 days of cell culture (Figure 5a). In the latter, cells seem to have invaded the formed membrane through channels (Figure 5c), formed perpendicularly to the borders of the capsule (Figure 5c). EPL‐labeled capsules were also characterized by confocal microscopy, in hydrated conditions, corroborating that the formed channels are formed through the whole inner membrane thickness, with a measured average diameter of 12 µm ± 3.6 µm (Figure 5d). Cell adhesion to the channels may be related to the exposure of EPL amine‐rich domains, previously correlated with the promotion of cell adhesion.^[^ ^34^ ^]^ A similar pattern was observed for 0.75 wt.% ALG capsules (Figure 5a; Figure S16, Supporting Information), namely for 15 min complexation. Interestingly, the presence of cells and their migration over time seems to have happened exclusively in the inner compartment of the 0.75 wt.% ALG capsules; no cells were detected or later invaded the outer compartment surrounded by the very thin wall characteristic of this formulation (Figure 5a; Figure S14a, Supporting Information). This suggests that cells mixed with the droplet phase are dragged upon the dehydration of the ALG + DEX phase during the formation of the second complexation region, which gives rise to the thicker and more robust inner membrane. Although localized rupture regions are observed during the formation of the multicore structures (Figure 3b; Movie S2, Supporting Information), cells are not released from the core of the 0.75 wt.% ALG capsules, suggesting the rapid formation of a semipermeable secondary inner membrane, which does not allow the passage of large micrometric objects as cells. The morphology of 0.50 wt.% ALG capsules comprising an outer membrane and a non‐spherical core was not observed after the addition of cells to the droplet phase (Figure S14b, Supporting Information). In fact, unicompartment and structures resembling the ones obtained 0.75 wt.% ALG cell‐free capsules were obtained in this case, regardless of complexation time. Therefore, the addition of cells to the droplet phase seems to influence the formation of the multicore structures, probably due to alterations in the overall viscosity of the droplet phase, or even due to the negative charge of cell surface, which may impact the interactions of EPL in the system. Capsules formed with 0.50 wt.% ALG did not enable cell adhesion and migration through channel‐like structures, which suggests that either these formulations do not enable the formation of channel‐like structures, or their internal diameter is too low to enable cell infiltration.

The rapid fabrication of multicompartment capsules may be useful for applications in drug delivery, cell culture, screening assays, and separation/retrieval of cells or molecules in low volumes. The technology reported here enables the spontaneous fabrication of structures that may selectively retain cargoes, and partially release fractions of encapsulated objects on demand. Interesting future work may comprise the study and tailoring of the permeability of different compartments to fabricate application‐targeted multicompartment devices or for mimicking living cells.^[^ ^35^ ^]^

## 3 Experimental Section

### Materials

Dextran *Leuconostoc spp*. (M_w_ 450 000–650 000 Da), sodium alginate from brown algae (M_w_ 120 000–190 000 g.mol^−1^), poly(ethylene glycol) (M_w_ 8000 Da), fluorescein isothiocyanate (FITC), dimethyl sulfoxide (DMSO, ≥99.5%), and phosphate‐buffered saline (PBS) pellets were purchased from Sigma‐Aldrich. ε‐poly‐L‐lysine (Epolyly Pure, M_w_ ∼ 4700 g.mol^−1^) derived from fermentation of *Streptomyces albulus* PD‐1 was obtained from Handary S.A (Brussels, Belgium). AlamarBlue cell viability reagent, and DiL staining (1,1′‐Dioctadecyl‐3,3,3′,3′‐Tetramethylindocarbocyanine Perchlorate) were purchased from Alfagene. For microgel encapsulation, calcium chloride anhydrous (CaCl_2_) and alginic acid sodium salt (M_w_ 10 000–600 000 g.mol^−1^) were obtained from PanReac. All reagents were used as received.

### Formation of Capsules

Solutions of 15 wt.% dextran (DEX) and 17 wt.% poly(ethylene glycol) (PEG) were prepared in PBS. Sodium alginate (ALG) was dissolved in the DEX 15 wt.% solution (Phase I), while  ε‐poly‐L‐lysine (EPL) was added to the 17 wt.% PEG solution (Phase II). The pH of Phase I and II was adjusted to 7.3‐7.4, after the complete dissolution of EPL. Uni‐ and multicompartment capsules were obtained using an electrohydrodynamic atomization equipment (Spraybase, Avectas) by applying and adjusting an electric voltage to 10 kV. Phase II was used as a collector bath and capsules were collected for 5 min, at an agitation of 350 rpm. Capsules were then left to complex for three distinct time periods, namely 2, 5, and 15 min, at 300 rpm in order to determine if the complexation time influences the capsule's morphology or compartment formation. Three washing cycles with PBS were performed to stop complexation and remove unreacted polymers, for 5 min, at 300 rpm. Flow was extruded at a rate of 10 mL h^−1^, through a 22G needle, and at a working distance (tip to collector distance) of 10 cm. Different ALG concentrations (0.50, 0.75, and 1.0 wt.%) and EPL concentrations (0.25, 0.50, 0.75, and 1.0 wt.%) were tested to promote interfacial complexation. The temperature of phase II solution was adjusted to be between 18 and 22 °C during extrusion.

Uni‐ and multicompartment capsules were also obtained with an airspray equipment (OB1 MK4 Microfluidic Flow Controller, Elveflow). Capsules were collected with a 26G co‐axial needle for 5 min at 350 rpm, with additional 5 min complexation, at an agitation of 300 rpm. An air pressure of 1500 mbar was kept constant.

### Capsules’ Diameter Measurements

30 to 175 measurements of the diameter of uni‐ and multicompartment capsules were performed in two independent assays using ImageJ image analysis software (NIH, USA) and a stereo microscope (ZEISS Stemi 508).

### Stability of Capsules in the Presence of NaCl

Capsules were obtained for 0.75 wt.% ALG + 0.50 wt.% EPL for 15 min of complexation as described previously in this section. Calcium chloride (NaCl) in a final concentration of 0.25 m was added to Phase II solution. Optical micrographs were acquired using a stereo microscope (ZEISS Stemi 508).

### Stability of Capsules at Different pH Values

The capsules were obtained for 0.50 wt.% ALG + 0.50 wt.% EPL, 0.75 wt.% ALG + 0.50 wt.% EPL wt.%, and 1.0 wt.% + 0.25 wt.% EPL for 5 min of complexation as described previously in this section. Afterward the capsules were exposed to either a PBS solution with pH adjusted to 5 or 9, using HCl or NaOH, respectively. Optical micrographs were taken using a stereo microscope (ZEISS Stemi 508).

### pKa Determination

The ionization constant (pKa) values for 0.75 wt.% ALG and 0.50 wt.% EPL in PBS were obtained by titration with 0.1 m HCl and 1 m NaOH, respectively. pH was monitored as small quantities (100 µL) of HCl and NaOH were added to each solution. The inflection points of the resulting titration curves were determined as pKa values.

### Labeling of EPL with FITC

EPL was dissolved in PBS at a concentration of 2 mg mL^−1^. After complete dissolution, pH was adjusted to 10.5 using droplets of 1 m NaOH. Another solution with FITC was prepared in DMSO with a concentration of 1 mg mL^−1^. To label EPL, the solutions were left to mix for 1 h at 4 °C in the dark. EPL‐FITC was precipitated using twice the volume with acetone and further dissolved in distilled water. Images of capsules produced using FITC‐EPL were acquired at 0 min (immediately after obtaining the capsules) and after three washes with PBS for 5 min under 300 rpm in a fluorescence microscope (Axio Imager M2, Carl Zeiss, Germany). Uni‐ and multicompartment capsules were visualized under a confocal microscope (LSM 900, Carl Zeiss, Germany) equipped with the ZEN Imaging software.

### Cell encapsulation in Core‐Shell Capsules

The cytocompatibility and cell arrangement in different capsules were assessed by using mouse fibroblast L929 cell line, obtained from European Collection of Authenticated Cell Cultures. L929 cells were cultured in DMEM‐LG (Dulbecco's modified Eagle medium, Sigma–Aldrich) supplemented with 10% fetal bovine serum (FBS) and 1% antibiotic/anitimicotic (ThermoScientific). Cells were used in passages between 47–50. Cell suspensions were prepared after trypsinization and incorporated in the Phase I. Three experiments were performed by using the following compositions: i) 15 wt.% DEX | 0.50 wt.% ALG + 17 wt.% PEG | 0.50 wt.% EPL, ii) 15 wt.% DEX | 0.75 wt.% ALG + 17 wt.% PEG | 0.50 wt.% EPL and iii) 15 wt.% DEX | 1.0 wt.% ALG + 17 wt.% PEG | 0.25 wt.% EPL. For cell encapsulation assays, the solutions used were sterilized: non‐equilibrated ATPS precursor solutions were filtrated using a sterile 0.2 µm pore filter (Whatman Puradisc Ø30 mm, Zmed), whereas ALG and EPL reagents were exposed to UV light for 40 min. Phase I and Phas were obtained after prior dissolution of sterile ALG and EPL, respectively. L929 cells at a cell density of 3 × 10^6^ cells mL^−1^ was resuspended in Phase I and uni‐ and multicompartment capsules were obtained using the aforementioned method. After 5 min of collecting capsules with a further specific complexation time of 2, 5, or 15 min, 3 washes in a 50 mL falcon with DPBS (30 mL) for 2 min each were performed and the final capsules were transferred to the appropriate cell culture medium. Cell encapsulated capsules were maintained at 37 °C in a humidified incubator with 5% CO_2_.

### Determination of Encapsulation Efficiency

After encapsulation, encapsulation efficiency was assessed by performing washes with PBS using only a few drops (no further washing) under a fluorescence microscope (Axio Imager M2, Carl Zeiss, Germany). Three experiments were performed by using the following compositions: i) 15 wt.% DEX | 0.50 wt.% ALG + 17 wt.% PEG | 0.50 wt.% EPL, ii) 15 wt.% DEX | 0.75 wt.% ALG + 17 wt.% PEG | 0.50 wt.% EPL and iii) 15 wt.% DEX | 1.0 wt.% ALG + 17 wt.% PEG | 0.25 wt.% EPL. L929 cells were used at a cell density of 3 × 10^6^ cells mL^−1^ and passage 47. Encapsulated cells were pre‐labeled with the lipophilic agent DiL, according to the manufacturer's instructions.

### Encapsulation in Continuous Alginate Hydrogels

L929 cells were encapsulated using 1 wt.% alginate in a bath of 0.1 m calcium chloride for 20 min. Both solutions were filtered with a sterile 0.2 µm pore filter (Whatman Puradisc Ø30 mm, Zmed). L929 cells were used at a cell density of 3 × 10^6^ cells mL^−1^ and passage 47. To achieve a similar size of the capsules obtained with the DEX | ALG + PEG | EPL system, hydrogels were collected with a 18G blunt needle for 5 min at 350 rpm, with additional 5 min complexation, at an agitation of 300 rpm. However, hydrogels with the same processing applied voltage parameters were also obtained as described previously in this section. Final hydrogels were transferred to the appropriate cell culture medium. Cell encapsulated hydrogels were maintained at 37 °C in a humidified incubator with 5% CO_2_.

### Cell Viability Assays

The viability of L929 cells within uni‐ and multicompartment capsules was evaluated at day 1, 3, 7, 14, and 21 days with a live/dead kit according with the manufacturer instructions (Invitrogen, USA). Encapsulated cells were incubated in cell culture medium with propidium iodide (PI) (Thermo Fisher Scientific) and Calcein‐AM solution (Thermo Fisher Scientific) at concentration of 1 and 2 mL mL^−1^, respectively, for 10 min at 37 °C. Encapsulated cells were washed with cell culture medium afterward and visualized in an upright widefield fluorescence microscope (Axio Imager M2, Carl Zeiss, Germany). A representative number of uni‐ and multicompartment capsules (*n* > 5) per independent experience were used for each assay at the different timepoints.

### Quantification of Metabolic Activity

The AlamarBlue assay (Thermo Fisher Scientific) was employed to evaluate the metabolic activity of encapsulated cells. Encapsulated cells were placed in a 24 well‐plate. AlamarBlueTM reagent was added to the cell culture medium in a 1:10 ratio for 17 h. Fluorescent measurements (λ_excitation_: 540 nm, λ_emission_: 600 nm) were conducted using a Synergy HTX microplate reader with a 96‐well black‐clear bottom plate. A representative number of 30 capsules or hydrogels were used for each assay at different timepoints.

### Statistical Analysis

Statistical analysis was performed using Graphpad Prism 8.0.1 and results are presented as mean ± standard deviation.

## Conflict of Interest

The authors declare no conflict of interest.

## Supporting information

Supporting Information

Supplemental Movie 1

Supplemental Movie 2

Supplemental Movie 3

## Data Availability

The data that support the findings of this study are available from the corresponding author upon reasonable request.
