# Supplementary material for: Spontaneous Formation of Solid Shell Polymeric Multicompartments at All‐Aqueous Interfaces
Source: Adv Sci (Weinh). 2024 Oct 4;11(45):2402592. doi: 10.1002/advs.202402592 (PMC11615791; doi:10.1002/advs.202402592)
Supplement: Supplementary file 1 — Supporting Information [file ADVS-11-2402592-s004.pdf]

ADVANCED SCIENCE
Open Access

Supporting Information

for *Adv. Sci.*, DOI 10.1002/advs.202402592

Spontaneous Formation of Solid Shell Polymeric Multicompartments at All-Aqueous Interfaces

Francisca G. Perfeito, Sara Vilabril, Andreia Cerqueira, Mariana B. Oliveira and João F. Mano**

Supporting Information for:

Spontaneous formation of solid shell polymeric multicompartments at all-aqueous interfaces

Francisca G. Perfeito[a], Sara Vilabril[a], Andreia Cerqueira[a], Mariana B. Oliveira[a*], João F. Mano[a*]

[a]*Department of Chemistry, CICECO – Aveiro Institute of Materials, University of Aveiro; Aveiro, 3810-193 Aveiro, Portugal.*

**Corresponding author. Email: Mariana B. Oliveira: mboliveira@ua.pt; João F. Mano: jmano@ua.pt*

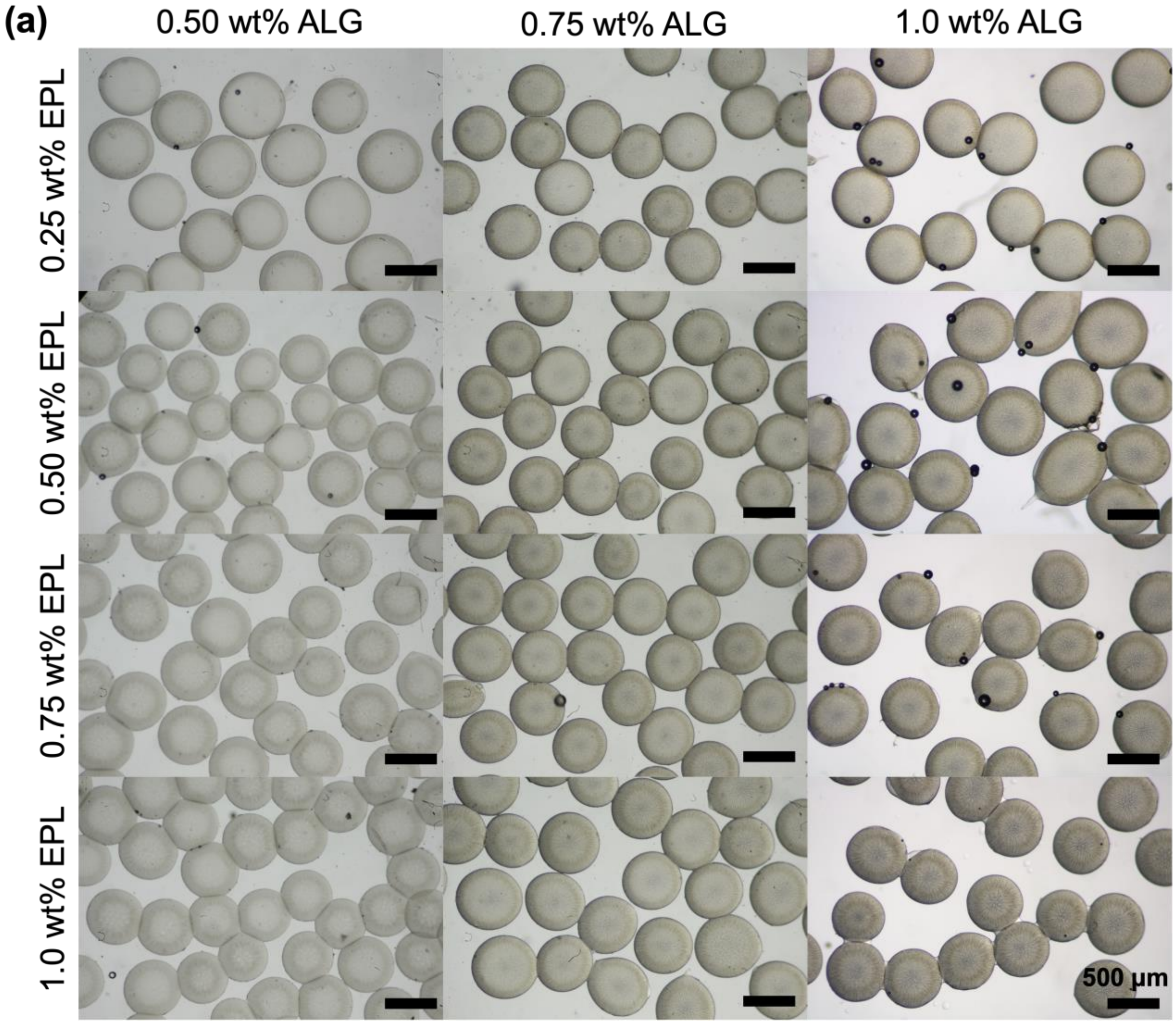

Fig. S1. Matrix of the uni- and multicompartment capsules obtained for 2 min of complexation before performing PBS washes.

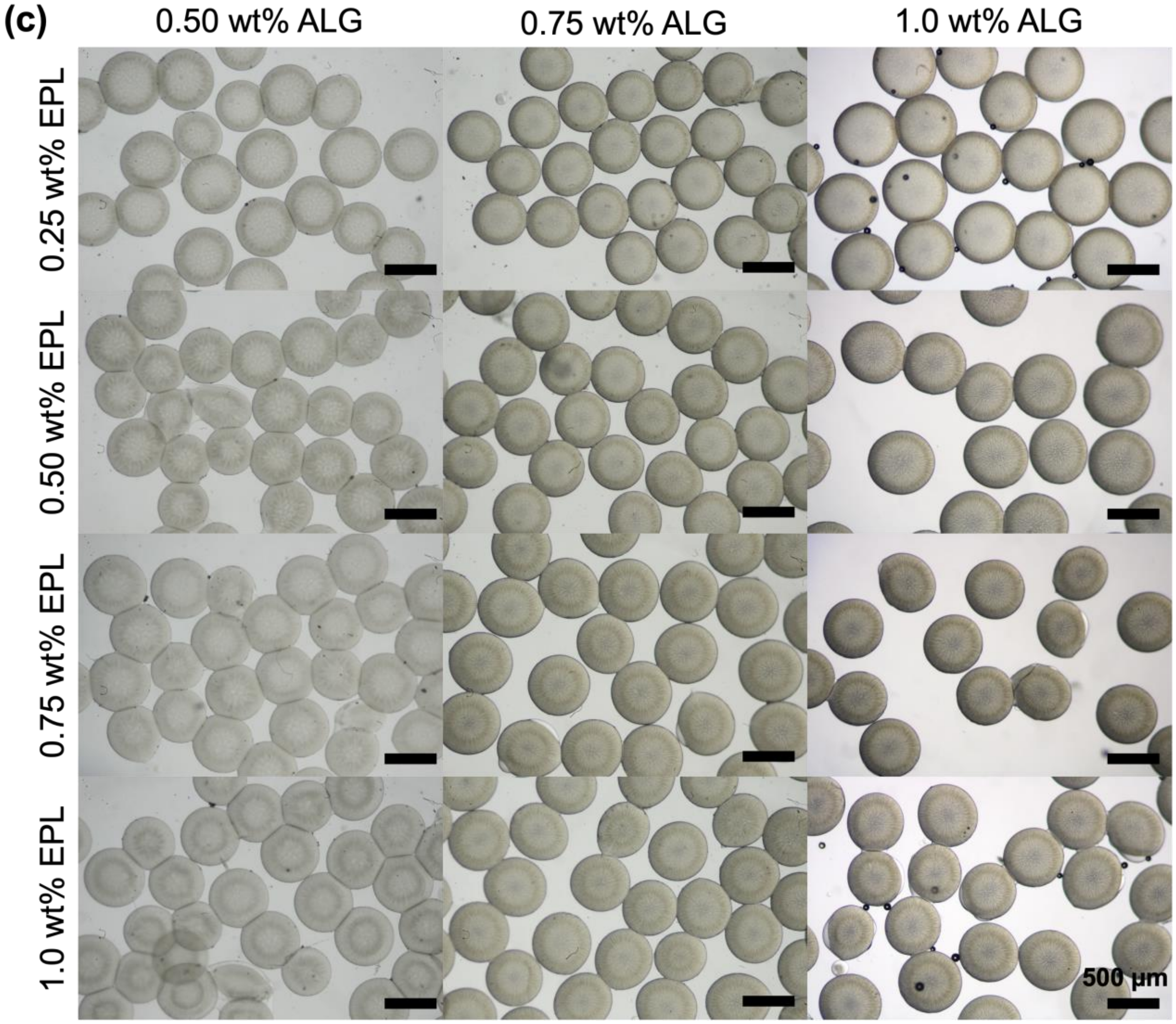

Fig. S2**.** Matrix of the uni- and multicompartment capsules obtained for 5 min of complexation before performing PBS washes.

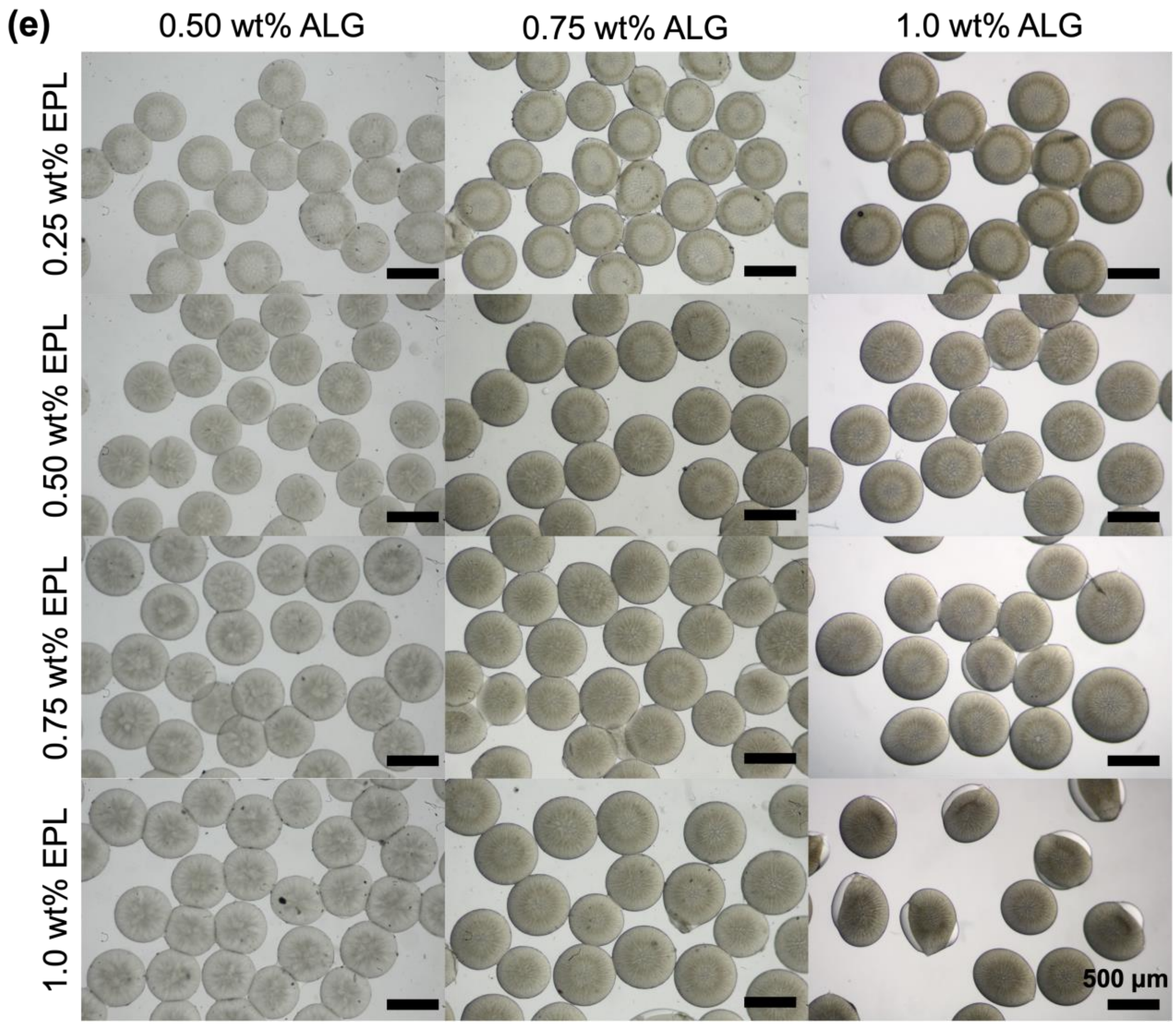

Fig. S3. Matrix of the uni- and multicompartment capsules obtained for 15 min of complexation before performing PBS washes.

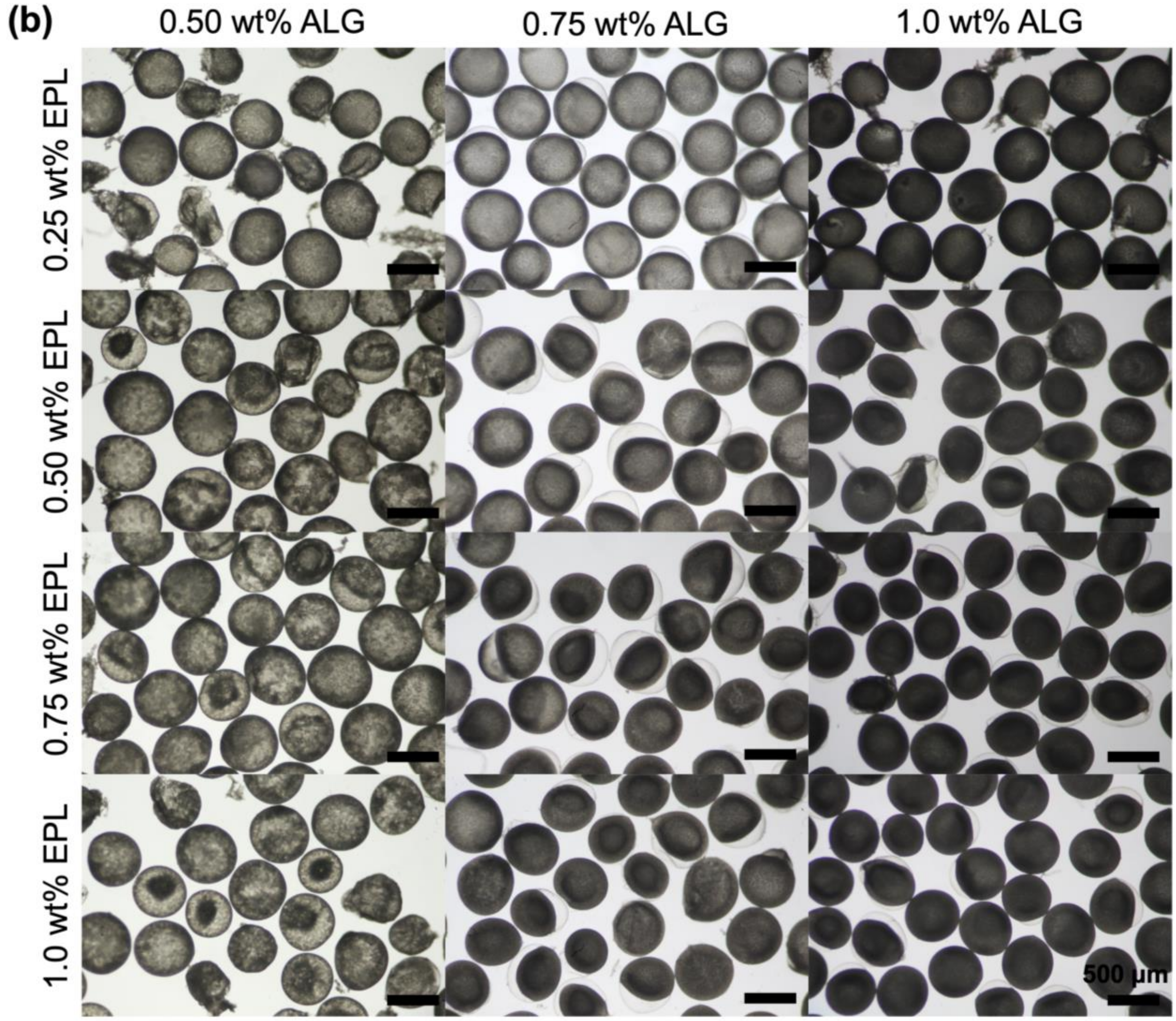

Fig. S4. Matrix of the uni- and multicompartment capsules obtained for 2 min of complexation after performing PBS washes.

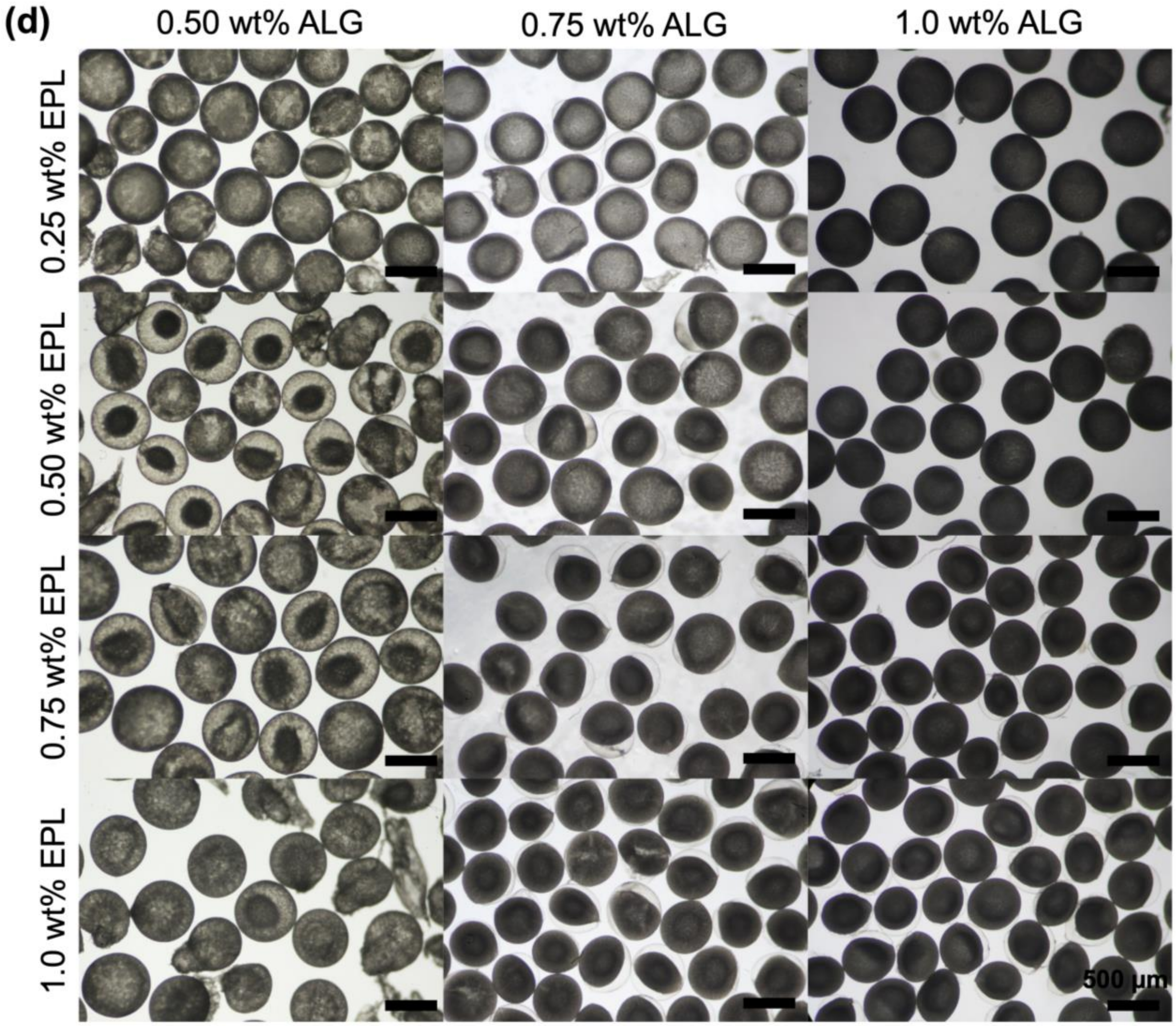

Fig. S5. Matrix of the uni- and multicompartment capsules obtained for 5 min of complexation after performing PBS washes.

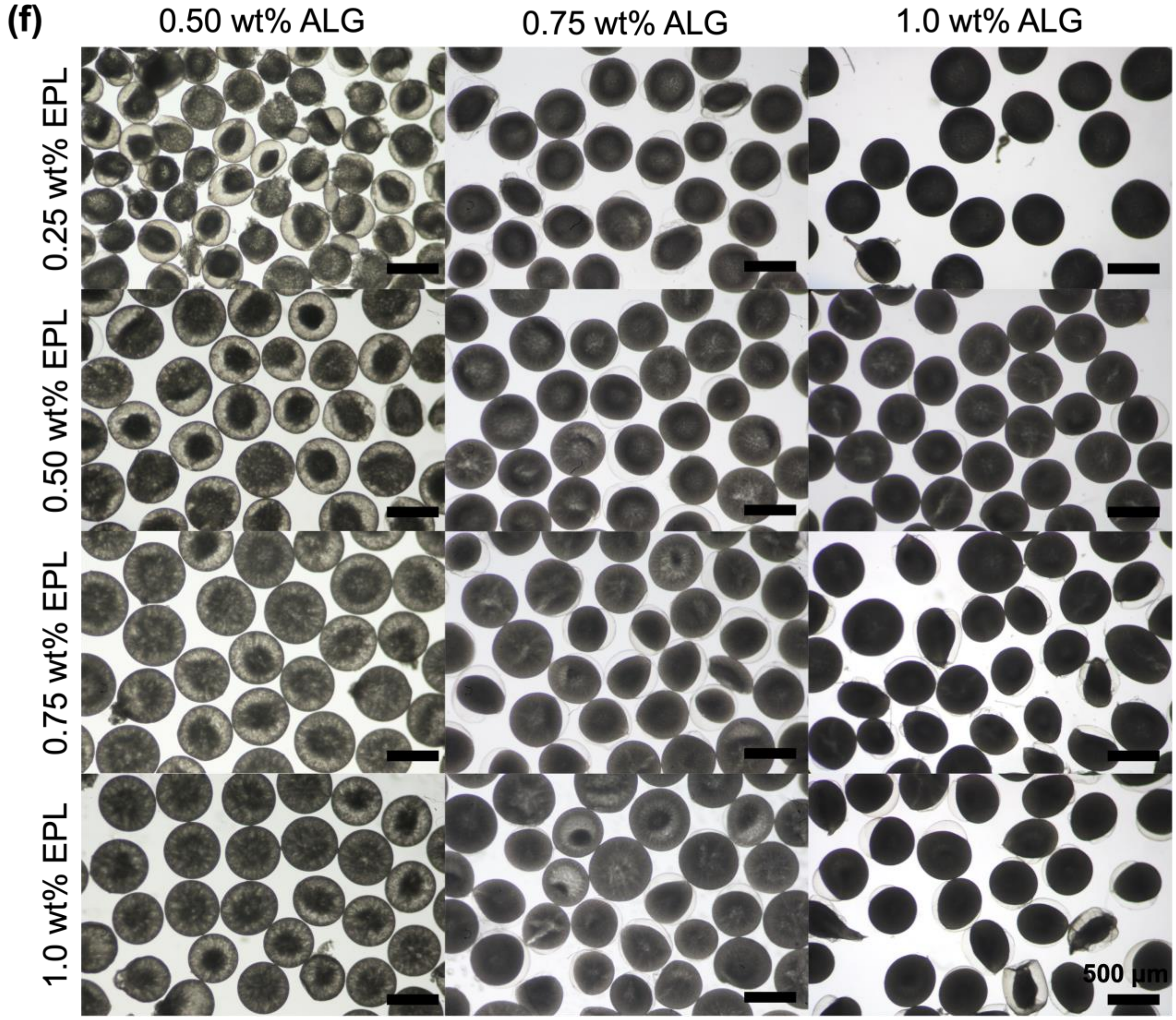

Fig. S6. Matrix of the uni- and multicompartment capsules obtained for 15 min of complexation after performing PBS washes.

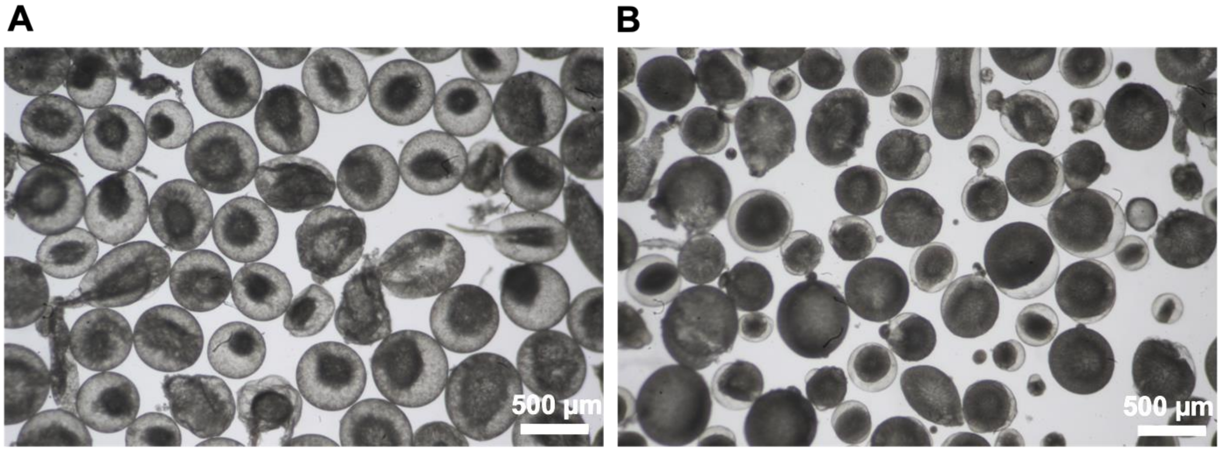

Fig. S7. Optical micrographs for the multicompartment capsules obtained with the air spray equipment. To obtain both capsules for (a) 0.50 wt% ALG + 0.50 wt% EPL, and (b) 0.75 wt% ALG + 0.50 wt% EPL, a pressure of 1500 mbar and a 26G co-axial needle at a constant magnetic agitation of 350 rpm.

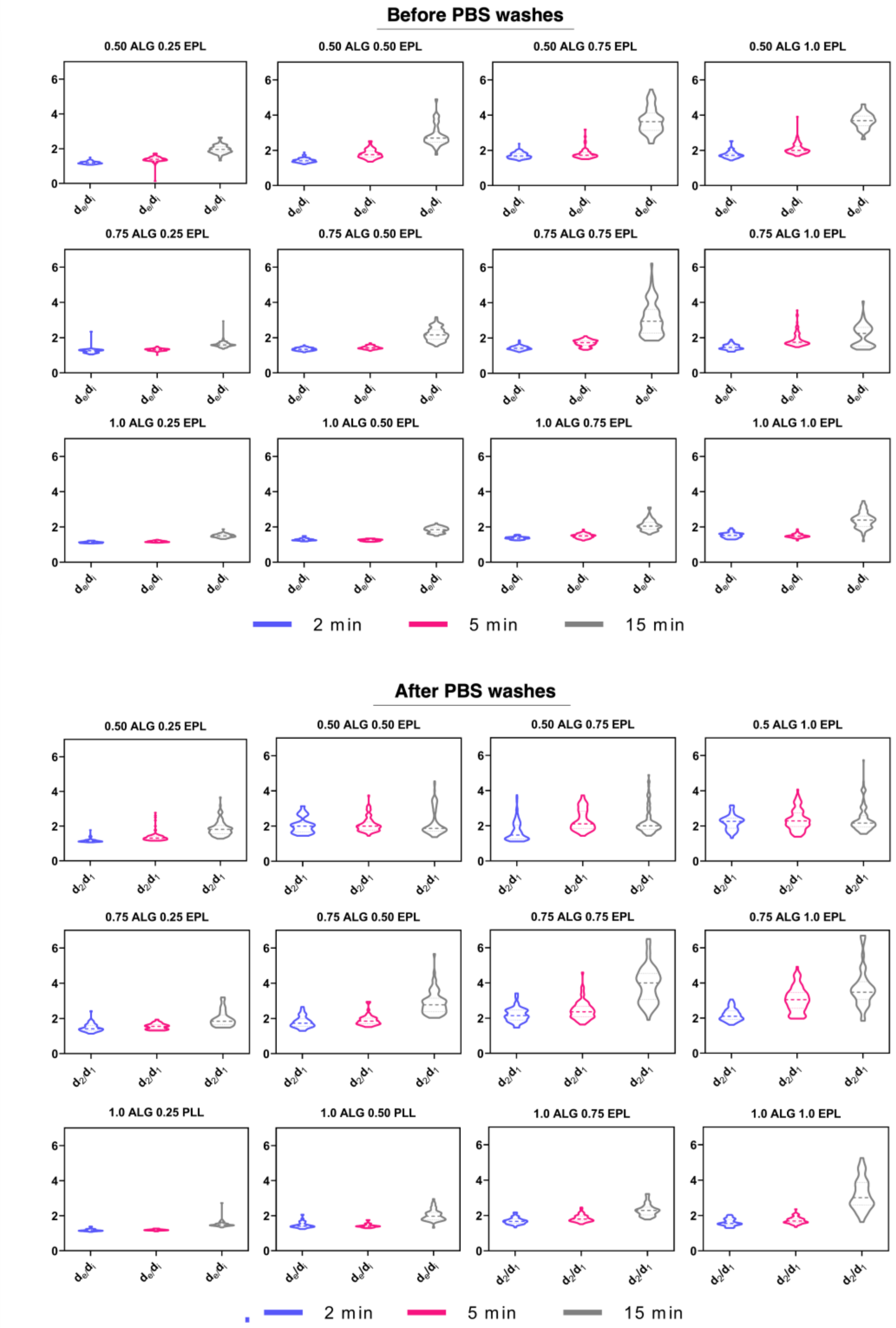

Fig. S8. Calculated ratio between the outer membrane diameter (d_e;d_2) and the inner membrane diameter (d_i;d_1) of the uni- and multicompartment capsules.

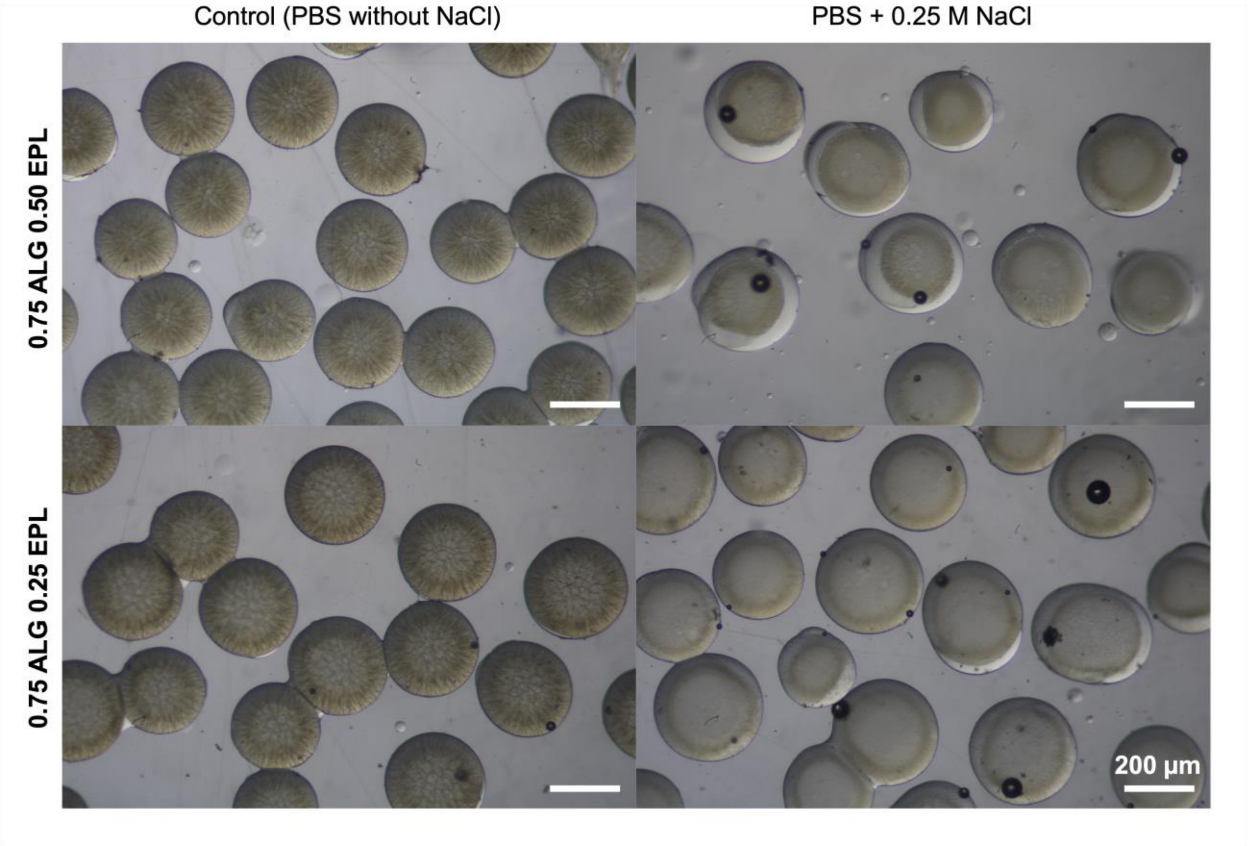

Fig. S9. Representative optical microscopy images of the multicompartment capsules obtained for 0.75 wt% ALG + 0.50 wt% EPL formulation, with 15 min complexation, without (left) or with (right) an additional 0.25 M of sodium chloride (NaCl) added to phase II. Data is presented for capsules before washing steps.

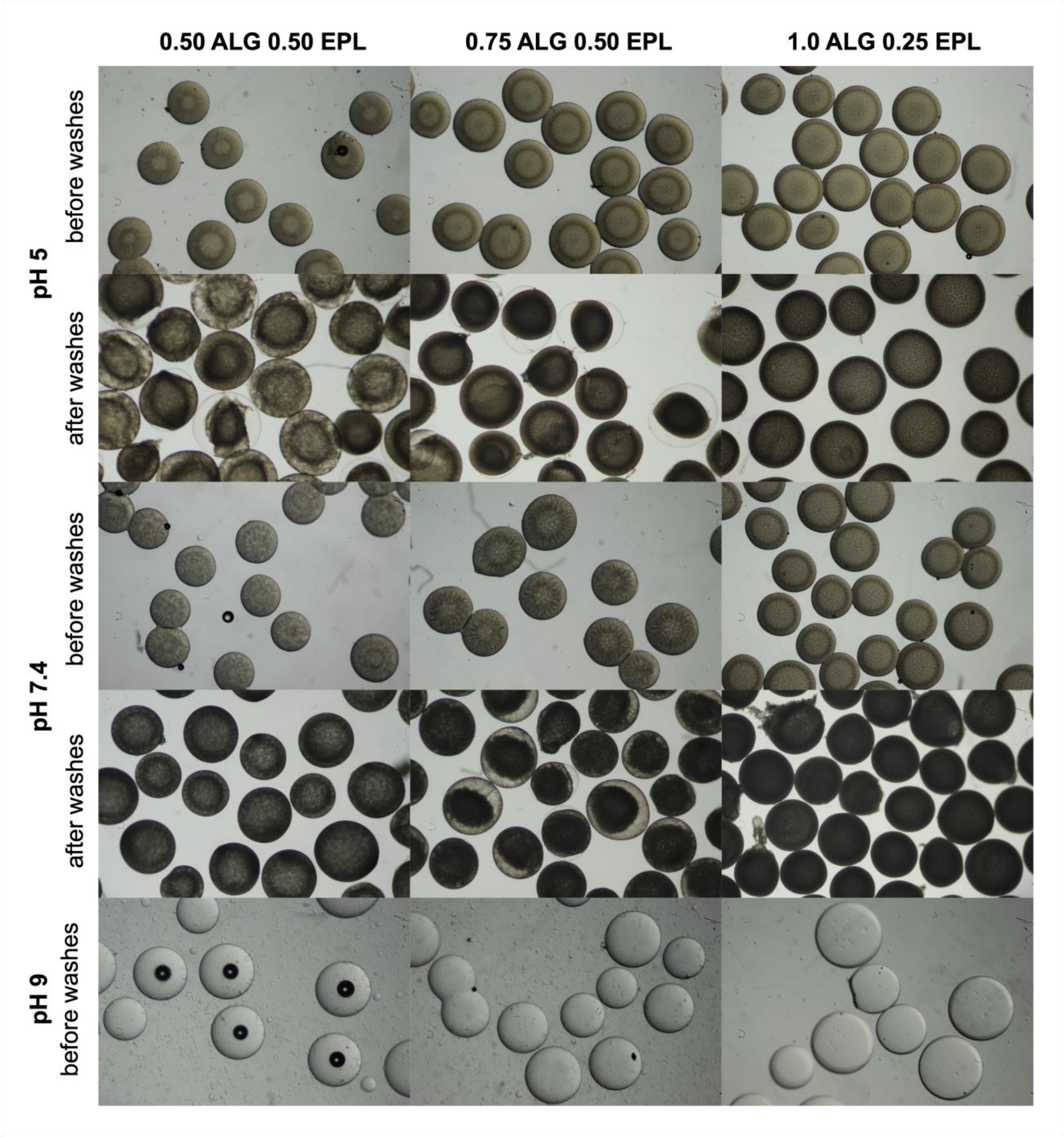

Fig. S10. Representative optical microscopy images of multicompartment capsules, for 15 min complexation, for phase II pH adjusted to pH of 5, 7.4 and 9. For pH 9, capsules disrupted on the 2nd wash with PBS (data not shown), demonstrating that for pH 9 less stable membranes are obtained.

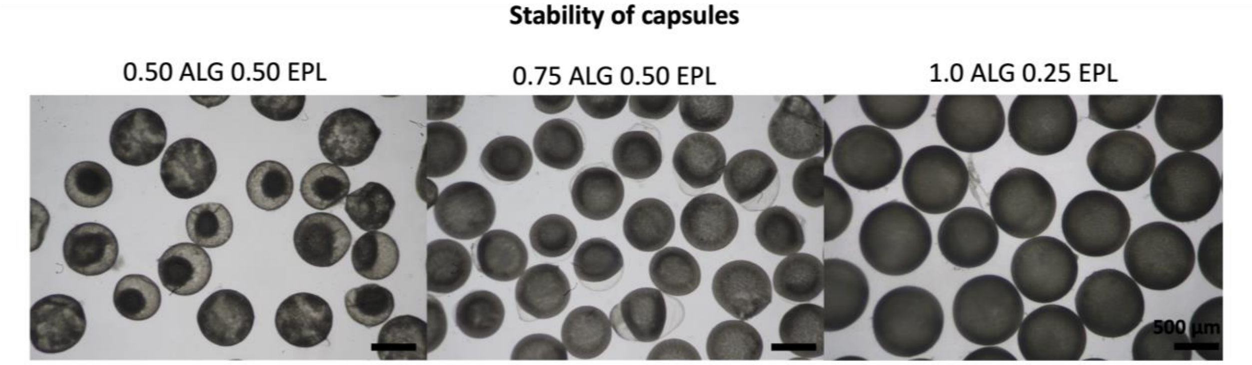

Fig. S11. Uni- and multicompartment capsule’s stability after 6 months stored in PBS at room temperature.

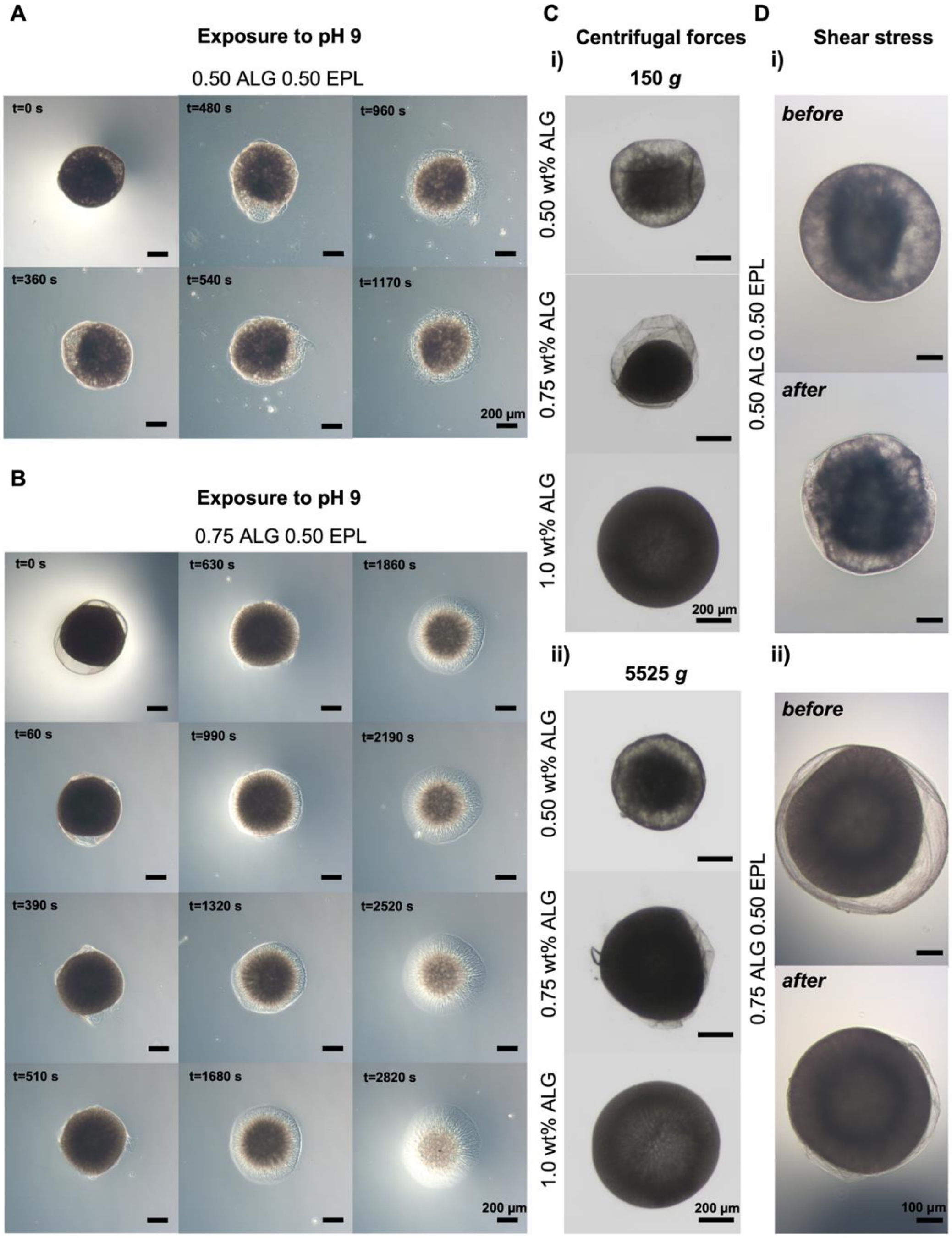

Fig. S12. Uni- and multicompartment capsule's behavior. (a) Exposure of 0.50 wt% ALG + 0.50 wt% EPL capsules obtained for 5 min of complexation to pH 9. (b) Exposure of 0.75 wt% ALG + 0.50 wt% EPL capsules obtained for 5 min of complexation to pH 9. (c) i) Capsules' stability obtained for 0.50, 0.75, 1.0 wt% ALG and 0.50 wt% EPL for 5 min at 150 *g*, and ii) 5525 *g*. (d) i) 0.50 wt% ALG + 0.50 wt% EPL capsules stability for shear stress induced with a 20 μm micropipette tip, ii) and for of 0.75 wt% ALG + 0.50 wt% EPL capsules.

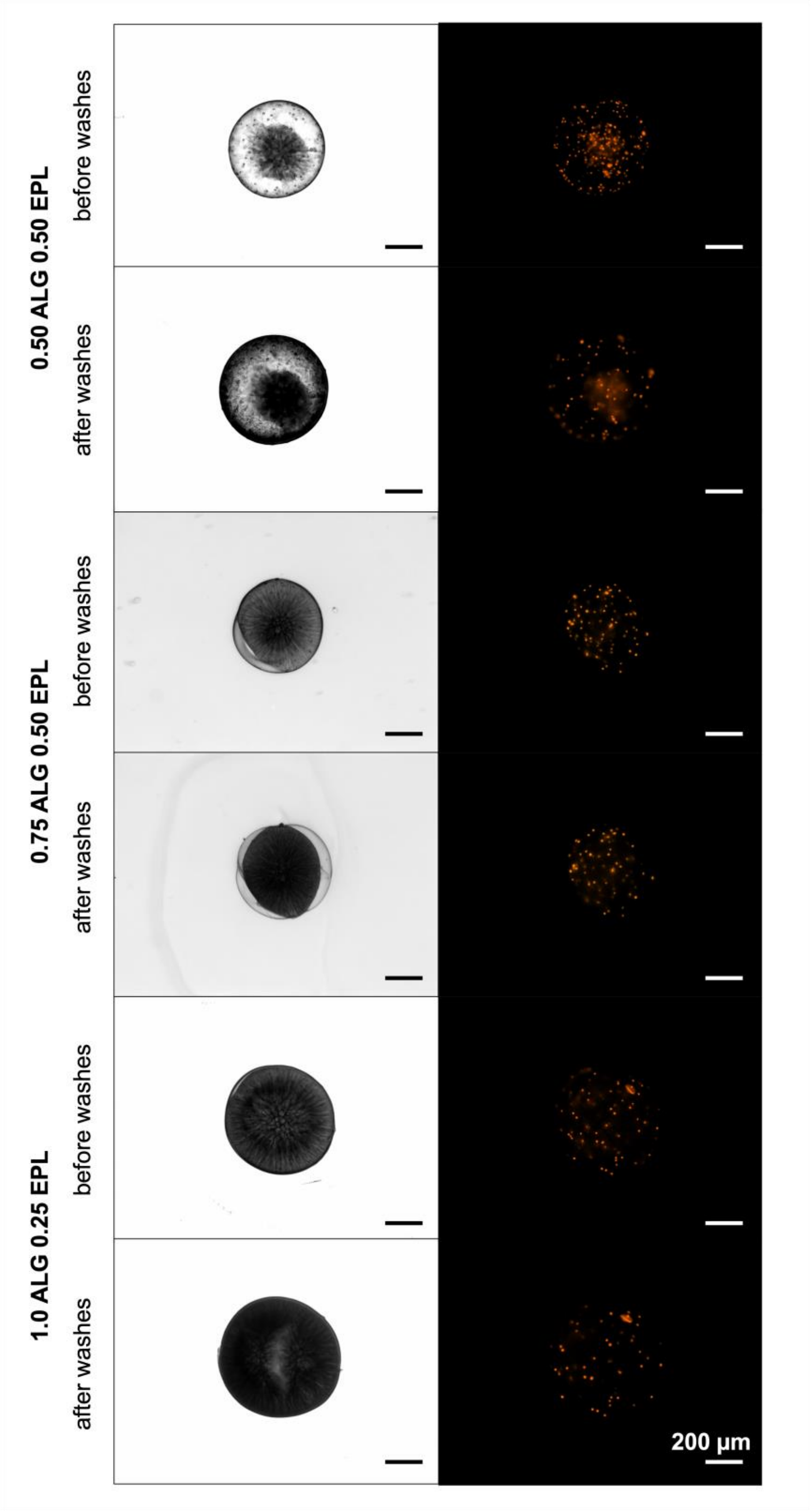

Fig. S13. Representative optical and widefield fluorescence microscopy of multicompartment capsules obtained for 5 min of complexation right after disruption with few drops of PBS (no further washing). Encapsulated cells were pre-labeled with the lipophilic agent 1,1'-Dioctadecyl-3,3,3',3'-Tetramethylindocarbocyanine Perchlorate (DiL).

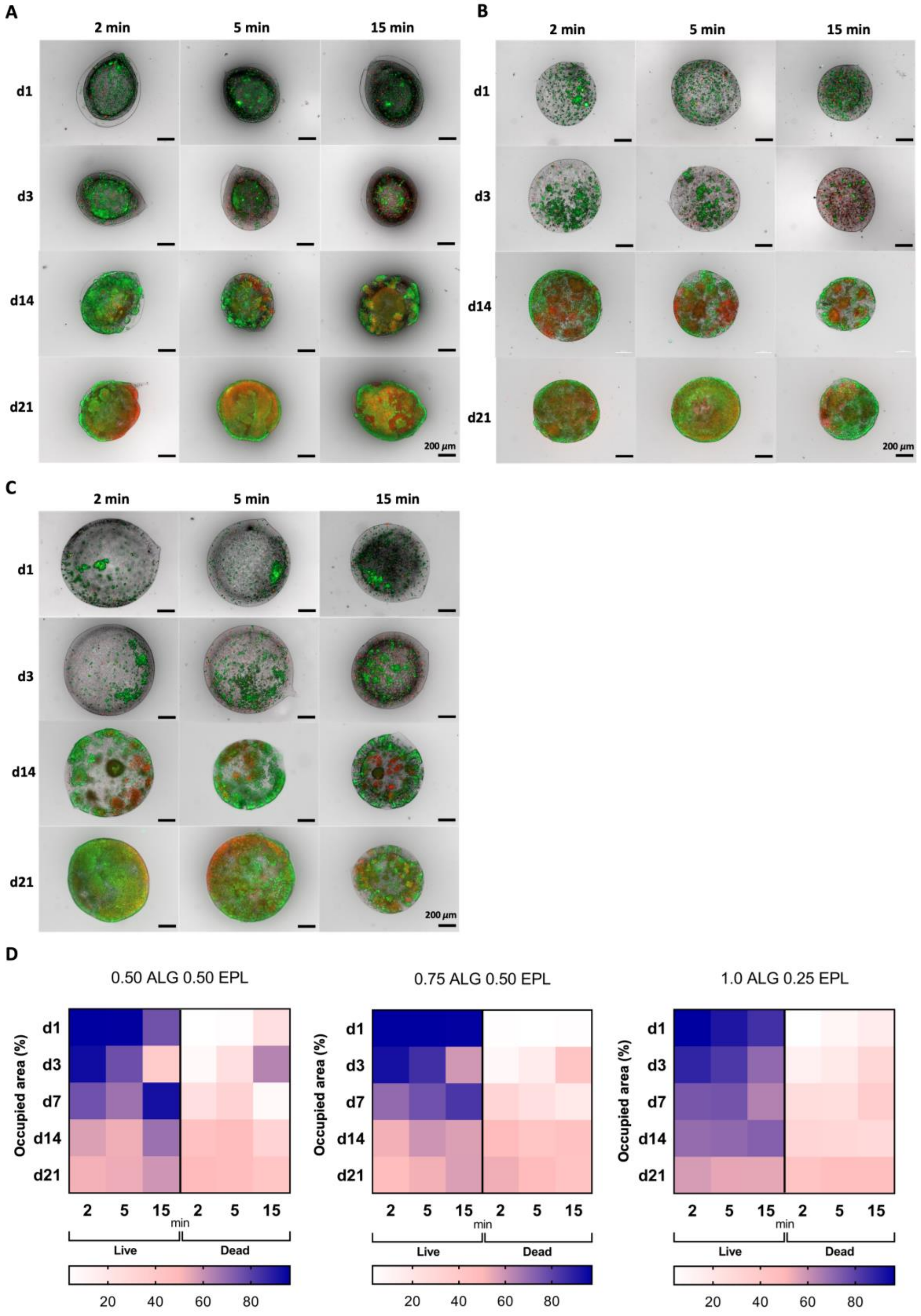

Fig. S14. (a) Live/Dead micrographs of multicompartment capsules for 0.75 wt% ALG after 1, 3, 14 and 21 days of culture. (b) Live/Dead micrographs of multicompartment capsules for 0.50 wt% ALG after 1, 3, 14 and 21 days of culture. (c) Live/Dead micrographs of unicompartment capsules for 1.0 wt% ALG after 1, 3,

14 and 21 days of culture. Staining - green: calcein-AM (live), red: PI (dead). (d) Heatmaps of the occupied area with cells for 21 days in culture.

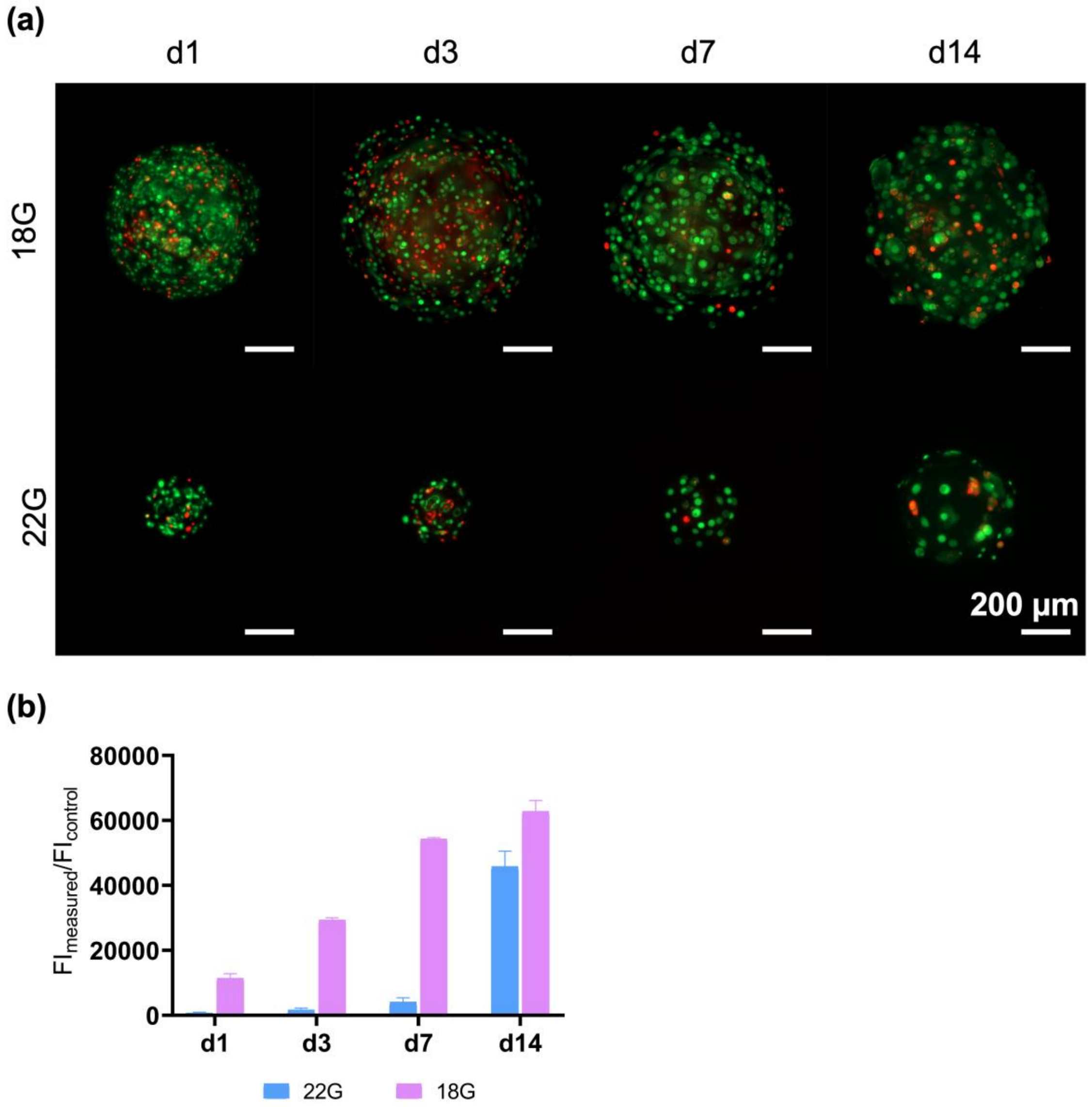

Fig. S15. (a) Hydrogels were obtained by complexation of 1% ALG in a 0.1 M calcium chloride ($CaCl_2$) solution for 20 min. A 18G and 22G blunt needle were used to prepare multicompartiment capsules with similar size to the counterpart core-shell capsules (18 G), or keeping the same applied voltage processing conditions (22 G). (b) The fluorescence intensity was measured using AlamarBlue assay (n = 1 independent experiment: 30 replicate hydrogels per well). The values of fluorescence in the hydrogels (Fl measured) were normalized with the fluorescence of DMEM cell culture medium (Fl control).

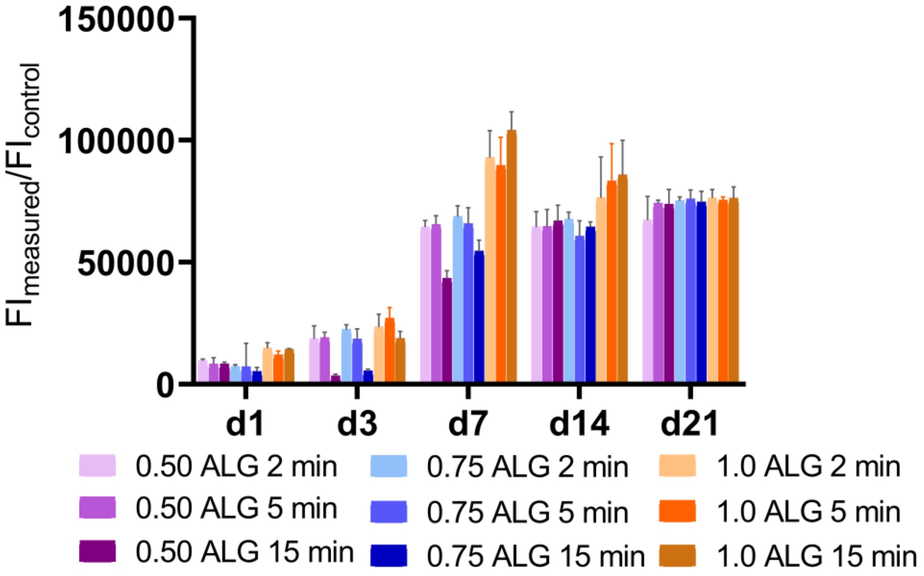

Fig. S16. Cell metabolic activity. The fluorescence intensity was measured using AlamarBlue assay (n = 3 replicates; 30 capsules per well). The values of fluorescence in all-aqueous uni- and multicompartment capsules (FI measured) were normalized with the fluorescence of DMEM cell culture medium (FI control). Average values, and error bars show the associated standard deviation value.

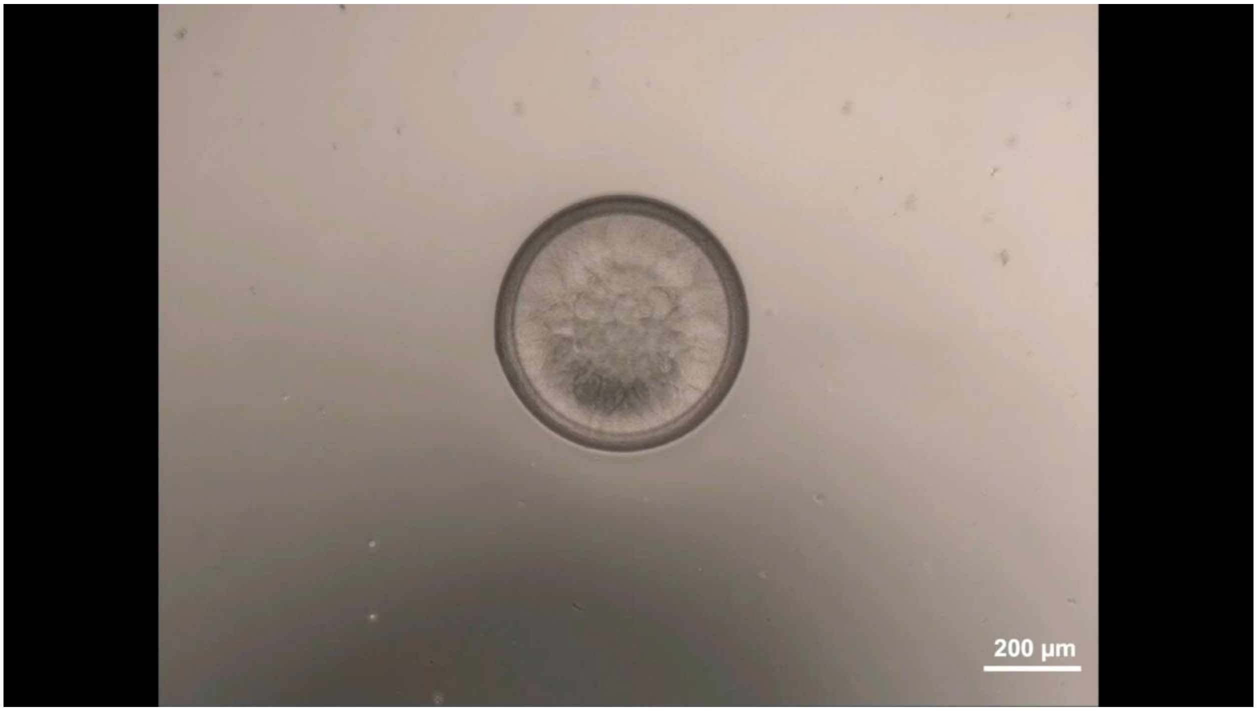

Movie S1. *In situ* washes with PBS for the 0.50% wt.% ALG capsule. The video was sped up 10x.

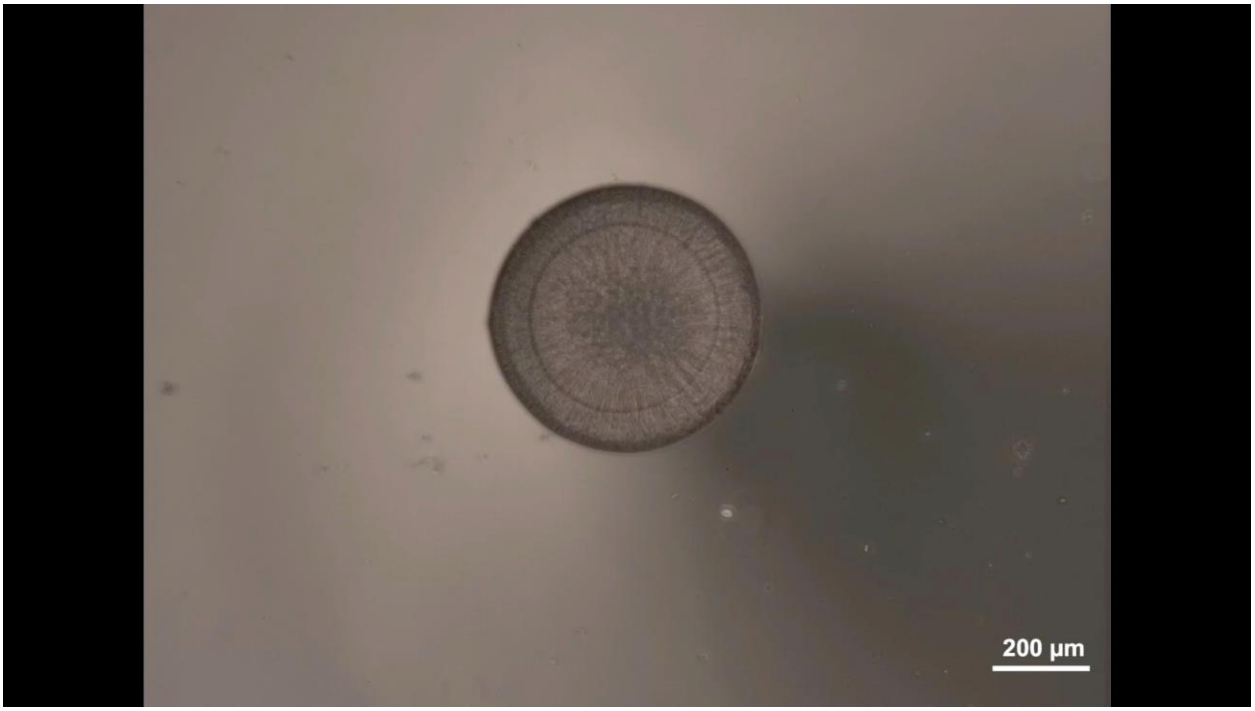

Movie S2. *In situ* washes with PBS for the 0.75% wt.% ALG capsule. The video was sped up 10x.

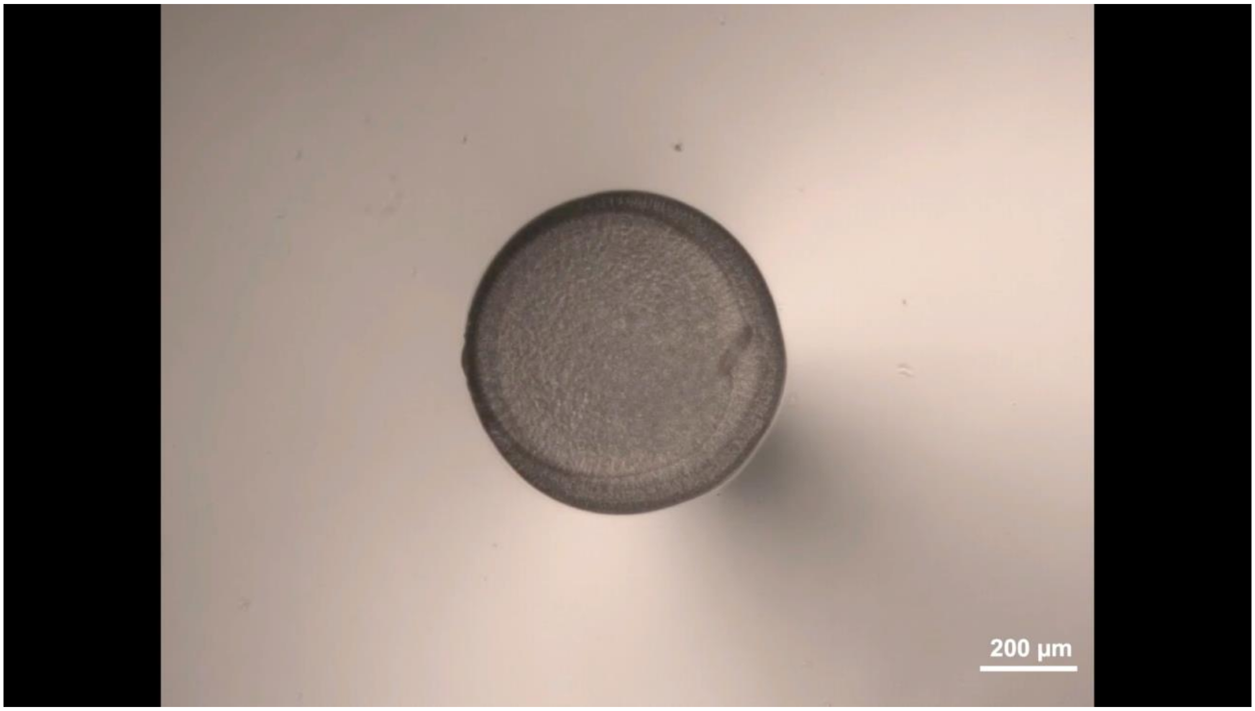

Movie S3. *In situ* washes with PBS for the 1.0% wt.% ALG capsule. The video was sped up 10x.
